# Thermal‐Assisted Multiscale Patterning of Nonplanar Colloidal Nanostructures for Multi‐Modal Anti‐Counterfeiting

**DOI:** 10.1002/advs.202305469

**Published:** 2023-10-22

**Authors:** Dan Su, Wei‐Long Wu, Pan‐Qin Sun, Yu‐Chen Yuan, Ze‐Xian Chen, Yun‐Feng Zhu, Kai‐Yu Bi, Huan‐Li Zhou, Tong Zhang

**Affiliations:** ^1^ Joint International Research Laboratory of Information Display and Visualization School of Electronic Science and Engineering Southeast University Nanjing 210096 China; ^2^ Key Laboratory of Micro‐Inertial Instrument and Advanced Navigation Technology Ministry of Education School of Instrument Science and Engineering Southeast University Nanjing 210096 China; ^3^ Suzhou Key Laboratory of Metal Nano‐Optoelectronic Technology Southeast University Suzhou Campus Suzhou 215123 China; ^4^ College of Software Engineering Southeast University Nanjing Jiangsu 210096 China

**Keywords:** multi‐modal anti‐counterfeiting, multiscale patterning, nano transfer printing, non‐planar colloidal nanostructures, self‐assembly

## Abstract

Nanotransfer printing of colloidal nanoparticles is a promising technique for the fabrication of functional materials and devices. However, patterning nonplanar nanostructures pose a challenge due to weak adhesion from the extremely small nanostructure‐substrate contact area. Here, the study proposes a thermal‐assisted nonplanar nanostructure transfer printing (NP‐NTP) strategy for multiscale patterning of polystyrene (PS) nanospheres. The printing efficiency is significantly improved from ≈3.1% at low temperatures to ≈97.2% under the glass transition temperature of PS. Additionally, the arrangement of PS nanospheres transitioned from disorder to long‐range order. The mechanism of printing efficiency enhancement is the drastic drop of Young's modulus of nanospheres, giving rise to an increased contact area, self‐adhesive effect, and inter‐particle necking. To demonstrate the versatility of the NP‐NTP strategy, it is combined with the intaglio transfer printing technique, and multiple patterns are created at both micro and macro scales at a 4‐inch scale with a resolution of ≈2757 pixels per inch (PPI). Furthermore, a multi‐modal anti‐counterfeiting concept based on structural patterns at hierarchical length scales is proposed, providing a new paradigm of imparting multiscale nanostructure patterning into macroscale functional devices.

## Graphical abstract

This work illustrates a thermal‐assisted non‐planar nanostructure transfer printing strategy for multiscale patterning of polystyrene nanosphere. The printing mechanism is the drop of Young's modulus, giving rise to an increased contact area, self‐adhesive effect, and inter‐particle necking. The multiscale colloidal nanostructure patterns on a 4‐inch wafer with over 2750 pixels per inch resolution enable a new concept of multi‐modal anti‐counterfeiting.

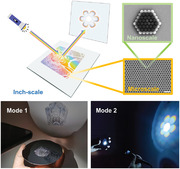

## 1 Introduction

Patterning nanostructures at different scales, such as metamolecules (consisting of two or more nanostructures), nanostructure superlattice, and metasurface have spawned the discovery of new physical mechanisms and applications.^[^ ^1^, ^2^, ^3^, ^4^ ^]^ Patterning at both the micro and macro scales bridges the nanostructures with particular optical, electrical, magnetic, and mechanical properties to functional macroscale devices.^[^ ^5^, ^6^, ^7^ ^]^

Large‐scale processing of patterned nanostructures remains a frontier problem in current nanofabrication research. Top‐down nanopatterning techniques, such as electron beam lithography, offer sub‐nanometer‐level single‐particle regulation^[^ ^8^, ^9^ ^]^ but face challenges in achieving 3D morphology control and large‐scale fabrication owing to high prices, complex operations, and long processing duration. In contrast, bottom‐up methods that employ atomic and molecular self‐assembly into nanostructures have made significant progress in morphology control and inter‐particle spacing control, with the advantages of large‐area assembly and low cost.^[^ ^10^, ^11^, ^12^ ^]^ However, the stochastic nature of the self‐assembly process makes it challenging to achieve deterministic patterning processing at large scales.^[^ ^13^, ^14^ ^]^ Recently, combinatorial strategies for bottom‐up colloidal nanostructure assembly driven by top‐down technology have been rapidly emerging, potentially enabling the modulation of nanoscale morphology, spacing, and deterministic patterning simultaneously.^[^ ^15^, ^16^, ^17^ ^]^ Among these methods, nanotransfer printing introduces a patterned stamp for the deterministic construction of nanostructures, offering advantages over controlled 3D morphology, nanoscale gap, low equipment requirements, and universal applicability to various substrates, opening up opportunities for the large‐scale fabrication of metamolecule,^[^ ^18^ ^]^ superlattice,^[^ ^19^, ^20^ ^]^ nanosolid crystal,^[^ ^21^ ^]^ and conformal colloidal pattern.^[^ ^22^ ^]^ However, constructing multiscale patterned nanostructure necessitates precise regulation of colloidal nanoparticles, metamolecules, and macroscale patterns, which is still challenging.

Theoretically, the interfacial transfer of nanostructure is dependent on the adhesion energy at the interfaces of nanostructure/polydimethylsiloxane (PDMS) stamp ($G_{c}^{\mathrm{Nanostructure}/\mathrm{PDMS}}$) and the nanostructure/receiving substrate ($G_{c}^{\mathrm{Nanostructure}/\mathrm{Substrate}}$).^[^ ^23^, ^24^ ^]^ The comparison of these two values determines the process of transfer printing, allowing for the identification of critical conditions necessary for pick up and printing.

For pick up

(1) $$G_{c}^{\mathrm{Nanostructure}/\mathrm{Substrate}}<G_{c}^{\mathrm{Nanostructure}/\mathrm{PDMS}}$$

For printing

(2) $$G_{c}^{\mathrm{Nanostructure}/\mathrm{Substrate}}>G_{c}^{\mathrm{Nanostructure}/\mathrm{PDMS}}$$

When it comes to planar nanostructures where the contact areas of Nanostructure/PDMS (S^Nanostructure/PDMS^) and Nanostructure/Substrate (S^Nanostructure/Substrate^) are almost the same, *G_C_* strongly correlates with both peeling velocity and temperature.^[^ ^25^, ^26^ ^]^ Successful printing of planar nanostructures such as nanodisk, nanoring, or nanoplate patterning has been realized using modulation of peeling velocity, temperature, or preload on the micron or larger scale.^[^ ^27^, ^28^, ^29^, ^30^ ^]^ However, regarding the nonplanar nanostructures, S^Nanostructure/PDMS^ is generally larger than S^Nanostructure/Substrate^, as the flexibility of the soft PDMS stamp induces a conformal contact. It is challenging to satisfy condition (2), resulting in low printing efficiency. Therefore, nonplanar nanostructure patterning is still elusive. Previously, transferring from the individual nanostructure scale to the micron scale was accomplished by introducing an adhesion layer with a thickness of ≈100 nm.^[^ ^31^ ^]^ However, the introduction of the adhesion layer complicates substrate processing and affects the optoelectronic properties of the nanostructures.^[^ ^32^ ^]^ Previous attempts included temperature‐controlled macro‐structure gripping without adhesive layers, which enabled the grip of macro 3D nonplanar objects and micro‐objects with sizes down to 10 µm.^[^ ^33^ ^]^ A novel method uses the dissolution of stamps by fully immersing them in an acetone bath for more than an hour, completely removing the adhesion of stamps to nanoparticles, and creating a pattern with a single particle resolution over a 10 µm scale.^[^ ^32^ ^]^ Nevertheless, multi‐scale nonplanar nanostructure patterning without an adhesive layer at both micro and inch scales is still lacking.

Here, we report a multiscale thermal‐assisted nonplanar nanostructure transfer printing (NP‐NTP) method of PS nanospheres and introduce the concept of multi‐modal anti‐counterfeiting through multiscale nanopatterning. By elevating the temperature to the glass transition point, we illustrate the reduction of the Young's modulus of PS nanospheres from 2500 MPa at ambient temperature to ≈30 MPa. The softer PS nanospheres possess an expansive contact area with the substrate and enhanced adhesion, yielding a substantial augmentation of printing efficiency to 97.2%. This method enables both micro and macro inch‐scale patterning with a printing efficiency of 97.2% and long‐range ordering. In addition, intaglio transfer printing (ITP) technology is utilized to create multiscale patterns up to 2750 PPI on a 4‐inch wafer for anti‐counterfeiting purposes. This study showcases a successful strategy for multiscale patterning of colloidal polymer nanostructures and introduces a novel anti‐counterfeiting concept that could inspire future research in multiscale nonplanar nanostructure patterning and transformative devices.

## 2 Results and Discussion

### 2.1 Multiscale Patterning of PS Nanospheres Using Thermal‐Assisted NP‐NTP

**Figure 1:**
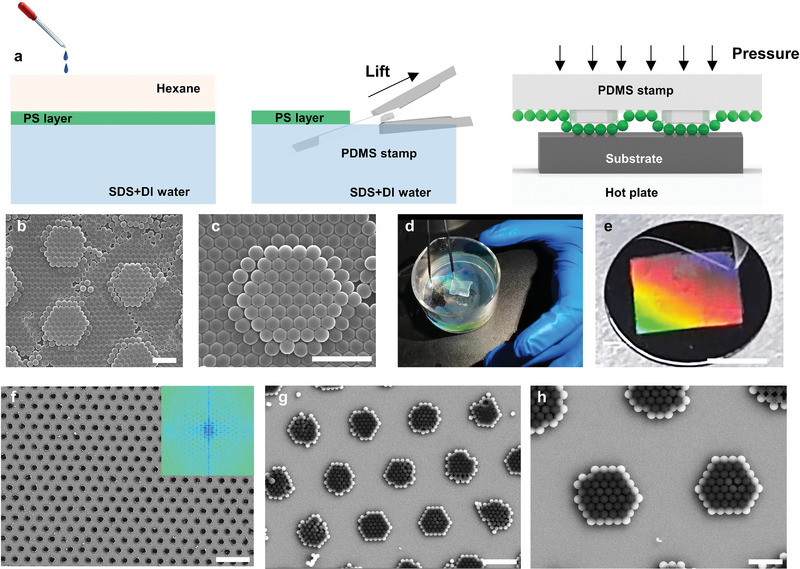
Multiscale nanopatterning with NP‐NTP. a) Schematic diagram of PS nanospheres NP‐NTP. PS layer was assembled at the water‐hexane interface and transferred using a PDMS stamp with the micro pattern. The pattern was then printed at a controlled temperature on a hot plate with proper pressure. b,c) Scanning electron microscopy images of PS nanosphere assembly transferred to PDMS. Scale bars, 2 µm. d) Photographs of transferring process of PS nanosphere layer at the water‐hexane interface. e) Photos of PS nanosphere layer printed to a silicon wafer. Scale bar, 1 cm. f–h) Scanning electron microscopy images of PS nanosphere assembly printed to the silicon wafer. The inset (f) is the corresponding FFT image. Scale bar, 20, 4, and 2 µm (f, g, and h, respectively).

The procedure to generate a well‐ordered multiscale PS nanosphere pattern is schematically shown in **Figure** **1**a. It consists of three sequential steps: two‐phase interfacial self‐assembly (step 1), Langmuir‐Blodgett (LB) transfer (step 2), and thermal‐assisted NP‐NTP (step 3).

In step 1, a hexane layer was introduced above the water to create a two‐phase interface. This interface facilitates the segregation of PS nanospheres toward the interface due to the Gibbs free energy reaching a minimum near the interface.^[^ ^22^ ^]^ Without hexane, the nanospheres would sink into the water and fail to assemble at the interface (Figure S1a‐d, Supporting Information). To induce compression and long‐range ordering of the nanospheres, sodium dodecyl sulfate (SDS) was added to increase the ion concentration (Figure S1e,f, Supporting Information).^[^ ^34^ ^]^ In addition, ethanol‐water mixed solution was used to disperse the PS nanospheres. The ethanol weakens electrostatic shielding, reduces the dielectric constant, introduces a surface tension gradient for the Marangoni effect,^[^ ^35^ ^]^ and thus promotes particle compression. The nanospheres tend to move toward the region with higher surface tension to reduce the surface energy.^[^ ^36^ ^]^ The limitation of the container wall causes the nanospheres to form a dense arrangement at the interface without any post‐processing. Refilling water is necessary to minimize the nanospheres dispersed in the aqueous phase (Figure S2, Supporting Information). The large‐area monolayer floating on the water surface exhibits a vivid peacock green color under omnidirectional illumination, indicating the uniformity and long‐range order of the assembled layer (Figure 1d). Compared to typical LB assembly method utilizing external driving forces^[^ ^37^ ^]^ and time‐consuming hydrophobization of nanoparticles (usually over 2 days),^[^ ^31^ ^]^ this process combined state‐of‐the‐art assembly ideas, can be achieved within 20 min without sophisticated equipment (Movie S1, Supporting Information) and has the potential to be applied to different materials as demonstrated in the assembly of SiO_2_ nanospheres (Figure S3, Supporting Information).

In step 2, the PS nanosphere layer was transferred to the oxygen plasma‐treated PDMS with pre‐designed micro patterns using the LB transfer method (Figure 1a; Figure S4, Supporting Information).^[^ ^38^ ^]^ Conformal single‐layer attained with the LB transfer method was verified by scanning electron microscopy (SEM) characterization (Figure 1b,c). Langmuir–Schaefer (LS) method is also widely adopted to transfer materials like Au nanoparticle,^[^ ^39^ ^]^ fibronectin molecules (FN),^[^ ^40^ ^]^ and GdF_3_ nanocrystals^[^ ^41^ ^]^ while it did not yield the desired results in our case. When the LS transfer method was used, multi‐layers were formed on the micro patterned regions of the PDMS stamp, which was different from what had been reported in other studies (Figure S5, Supporting Information).^[^ ^41^ ^]^ This result was attributed to the air layer that formed between the PDMS stamp and the PS layer during transfer, which prevented conformal contact between the two surfaces. Under this circumstance, inter‐particle adhesion and capillary force of the water leads to aggregation and multi‐layer (Figure S6a, Supporting Information). When LB transfer method is used, the PS layer and the stamp surface come into contact gradually during evaporation of the water layer, maintaining the integrity and long‐range order of the PS layer (Figure S6b, Supporting Information).

In step 3, thermal‐assisted NP‐NTP was conducted under proper pressure and glass transition temperature of the PS nanosphere (Figure S7, Supporting Information). The resulting multiscale pattern is shown in Figure 1e–h, which demonstrates successful printing of macro‐scale patterns in addition to highly uniform and colorful nanostructures on a silicon wafer. The pattern shows good conformality on individual metamolecules and maintains uniformity and periodicity over ≈1 inch, as confirmed by fast Fourier transform analysis (FFT) (Figure 1f). Uniform pressure during the printing process is an essential factor in ensuring the quality of the pattern, which is consistent with previous literature.^[^ ^26^ ^]^ The thermal‐assisted NP‐NTP offers three distinct advantages. First, the process is simple that does not necessitate etching or dissolving templates, making it applicable in printing organic nanomaterials.^[^ ^32^ ^]^ The printing procedure takes only ≈5 min while some previously reported strategy takes more than an hour or needs a vacuum transferring environment.^[^ ^42^, ^43^ ^]^ Second, the thermal‐assisted NP‐NTP does not require any complex or precise equipment, further extending its potential applications.^[^ ^26^, ^44^ ^]^ Lastly, the process does not require an additional adhesive layer, which mitigates the risk of interference with the optical properties resulting from factors such as alterations in the refractive index.^[^ ^26^ ^]^

**Figure 2:**
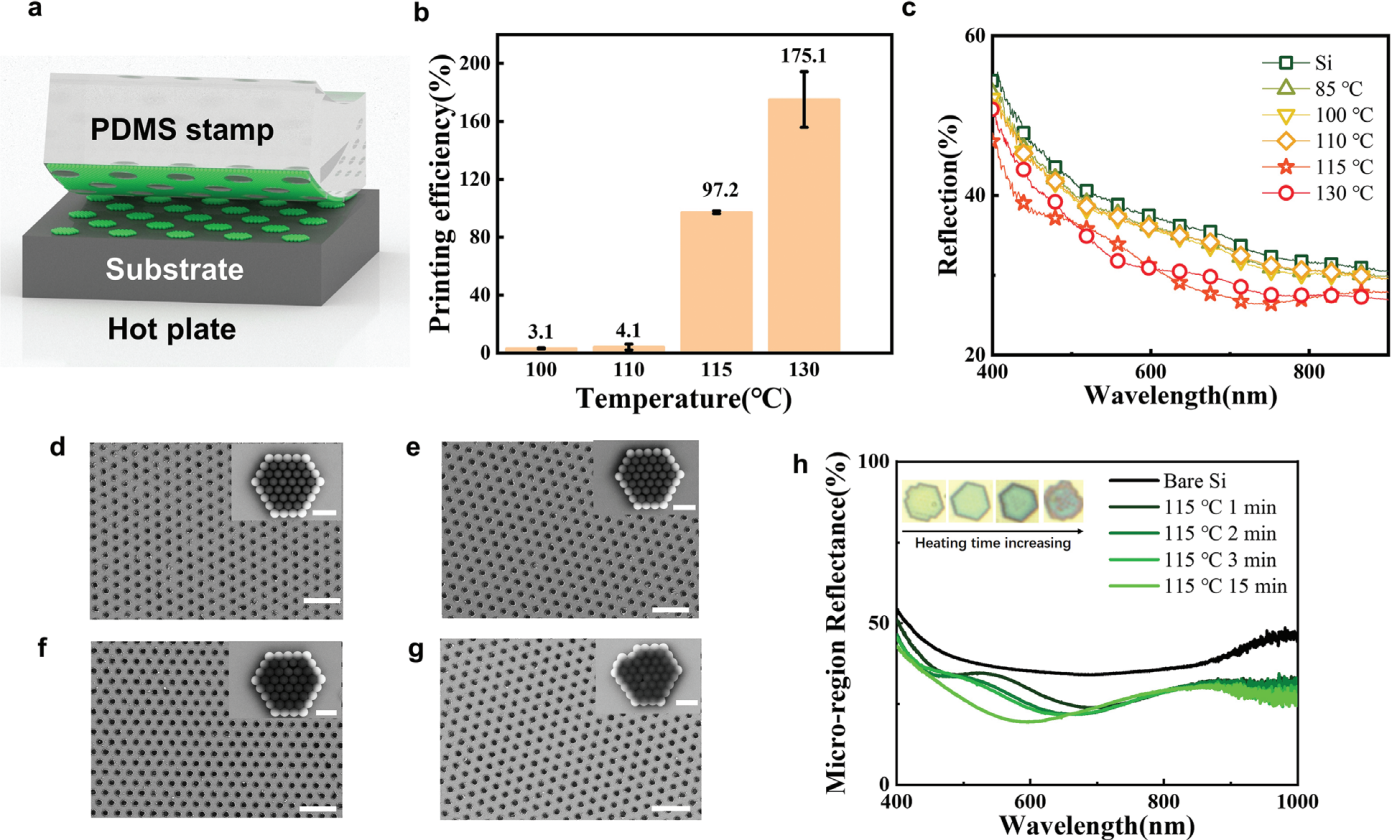
Temperature‐dependent printing efficiency and order of PS nanopatterns attained with NP‐NTP. a) Schematic diagram of periodic and long‐range‐ordered micro pattern printed. b) Printing efficiency at different printing temperatures. c) Reflectance spectra of the printed PS metamolecule arrays on a silicon wafer at different temperatures. d–g) Scanning electron microscopy images of PS nanosphere assembly heated for different durations of 1, 2, 3, 15 min, respectively. Scale bar, 1 µm. h) Estimated micro‐region reflectance of PS nanosphere films on a silicon wafer. The inset shows microscopic optical images of the micro pattern at the corresponding heating duration.

We point out that it is critical to print at the glass transition temperature of the PS nanosphere. Temperature‐dependent printing experiments were conducted to verify the significance of this crucial parameter. **Figure** **2**a illustrates the ideal conformal printing condition. It is noticed that the PS micro pattern is not in exact accordance with the circular pattern of the stamp. A hexagonal arrangement within a PS metamolecule is observed because the size of the nanosphere is comparable to the pattern and is not negligible (Figure 1c). To characterize the printing efficiency of the printing process, we randomly selected various regions from samples heated at different temperatures and for different transfer duration for statistical analysis. The calculation of the printing efficiency is based on the following formula^[^ ^22^ ^]^:

(3) $$\mathrm{Printing}\;\mathrm{efficiency}\;=S_{\mathrm{printed}}\;/S_{\mathrm{standard}}$$

where *S*_printed_ and *S*_standard_ represent the actual and ideal area of printed micro patterns on a silicon substrate. The printing efficiency undergoes a drastic increase at 115 °C, jumping from ≈3% to ≈97.2% (Figure 2b). The reflectance spectrum also decreases with the increase in printing efficiency in the range of 100–115 °C. In the case of 130 °C, the reflectance valley shifts to a longer wavelength (Figure 2c). This can be attributed to the complete fusing of nanospheres, indicated by SEM images. The nanospheres are arranged in a regular quasi‐hexagonal pattern at 115 °C, while the nanospheres printed at 100 and 110 °C appear randomly scattered and irregular (Figure S8, Supporting Information). In the case of 130 °C, obvious residuals were observed between the micropatterns. This results in an unwanted printing efficiency of over 100% and a low reflectance at the expense of the conformality of the array (Figure 2c).

We further show that when heated at 115 °C, color and micro‐region spectra modulation can be achieved by controlling printing duration. At this temperature, printing efficiency exceeding ≈90% can be achieved within 1–15 min, with long‐range ordering ensured (Figure 2d–g; Figure S9, Supporting Information). The results of micro‐region imaging and the spectra show that the color of the micro‐pattern can be changed from yellow to green and even dark green by controlling the printing duration (Figure 2h). This is due to the limited increase of fusing between spheres at different heating duration, which is different from cases of 130 °C, brings new degrees of freedom of control for optics properties.

### 2.2 Principle of Thermal‐Assisted NP‐NTP

**Figure 3:**
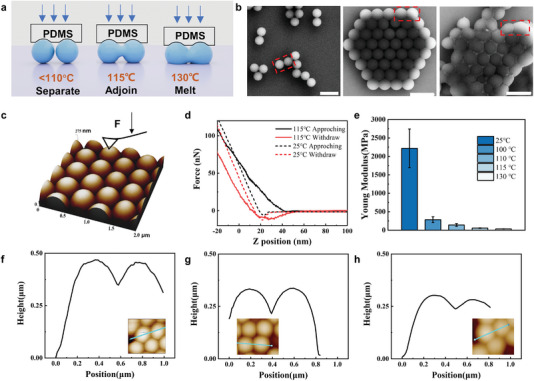
Viscoelasticity mediated printing mechanism around glass transition temperature. a) Schematic diagram of the printing and the interactions between PS nanospheres, PDMS stamp, and receiving substrate at different printing temperatures. b) SEM images of PS nanospheres treated by different temperatures, from left to right: 100, 115, 130°C. Scale bar is 1 µm. c) 2 × 2 µm AFM image of closed‐packed PS nanosphere and AFM tip indentation characterization, during which the AFM probe was placed above the center of the nanosphere and slowly lowered while drawing its force–displacement. d) Typical force–displacement curves of PS nanosphere under 25 and 115 °C. e) Young's modulus fitting by Hertz model derived from variable temperature‐dependent AFM force–displacement approaching curves. f–h) Height distributions at 100 °C (left), 115 °C (middle), and 130 °C (right). The horizontal coordinates increase in the direction of the blue line from left to right.

The underlying mechanism of the temperature‐dependent PS nanosphere printing process is illustrated in **Figure** **3**a. SEM images at different temperatures reveal that when the printing temperature is below 110 °C, the nanostructures are randomly distributed, separated from each other, and no pattern is formed (Figure 3b). At 115 °C, metamolecule micro patterns form as the nanospheres begin necking with each other. Further increasing the temperature to 130 °C leads to significantly elevated fusing effects between the PS nanospheres, noticeable nanosphere deformation, and blurred inter‐particle boundaries. The thermal‐assisted NP‐NTP mechanism depends on three possible contributions: i) thermal softening of PS nanospheres increases the contact area and resulting in larger a larger $G_{c}^{\mathrm{PS}/\mathrm{Substrate}}$, ii) the mobility of molecular chains enhances viscosity at the glass transition temperature, creating a larger adhesion force, iii) necking between nanospheres driven by temperature and external forces creates a region conducive to high‐efficiency nanopattern printing.

In the temperature range at room temperature or significantly below the glass transition temperature, the Young's modulus of the PS nanosphere (≈2 GPa) is much larger than that of the PDMS stamp (≈10 MPa), while still smaller than that of the silicon substrate (100–200 GPa).^[^ ^45^, ^46^ ^]^ That is to say, the PDMS stamp is the softest, making it easier to reshape. Even under considerable force, the contact area of PS nanospheres and the silicon substrate is smaller than that of PS nanospheres and PDMS stamp, hindering the printing process. This is contrary to the results of the transfer criterion for film adhesion energy, which according to the literature $G_{c}^{\mathrm{PS}\;\mathrm{film}/\mathrm{PDMS}}$= 50 mJ m^−2^,^[^ ^47^, ^48^ ^]^ while $G_{c}^{\mathrm{PS}\;\mathrm{film}/\mathrm{Si}}$= 1 J m^−2^.^[^ ^49^, ^50^ ^]^ Although $G_{c}^{\mathrm{PS}\;\mathrm{film}/\mathrm{Si}}$>$G_{c}^{\mathrm{PS}\;\mathrm{film}/\mathrm{PDMS}}$ for 2D film architectures, the adhesion between PS nanospheres and substrate is exceptionally weak due to the extremely small contact area of nonplanar nanospheres, resulting in a low transfer printing efficiency of ≈3%. We point out and clearly demonstrate that the kinetically controlled transfer printing method and the model are not applicable to non‐planar nanostructures. However, this issue is frequently disregarded in contemporary research on nonplanar nanostructures, leading to a diminished reliability of the printing method, featuring a substantial variation in printing efficiency.^[^ ^22^ ^]^

As the temperature gradually rises to the glass transition temperature, the Young's modulus of the PS nanospheres decreases significantly (Figure 3e). This decrease in Young's modulus makes the nanospheres more susceptible to deformation when in contact with the substrate. To better understand the printing mechanism, temperature‐dependent Young's modulus of PS nanospheres was characterized by atomic force microscopy (AFM) tip indentation at different temperatures. (Figure 3c; Figure S10, Supporting Information). Specifically, the large slope in the approaching force‐displacement curve characterizes the large Young's modulus (Figure 3d). As the temperature increases to the glass transition temperature, the Young's modulus of PS nanospheres decreases significantly from 2500 to ≈30 MPa, which is only ≈12% at room temperature. To further investigate the contact area of PS nanospheres and Si substrate, a spherical approximation was applied to estimate the contact area from the height curves (Figure S11, Supporting Information). As the temperature increases, the height of the PS nanosphere decreases to 0.468 µm (100 °C), 0.323 µm (115 °C), and 0.300 µm (130 °C), respectively (Figure 3f–h), which yields an increase of PS nanosphere‐silicon contact area of 0.0011, 0.1420, 0.1589 µm^2^ while the upper semi‐sphere retains an almost unchanged shape, attributable to the conformal contact between the soft PDMS stamp and nanospheres. A larger contact area means larger adhesion force and adhesion tunability, making the adhesion energy of nonplanar nanostructures closer to that of 2D film interface. Therefore, the printing condition is satisfied with $G_{c}^{\mathrm{PS}\;\mathrm{nanospheres}/\mathrm{Si}}$>$G_{c}^{\mathrm{PS}\;\mathrm{nanospheres}/\mathrm{PDMS}}$, contributing to high‐efficiency printing.

The main influencing factors in the printing process are the van der Waals force and electrostatic force at the PS nanospheres/receiving substrate interface. The viscosity of the PS nanospheres is significantly increased due to the overall effect of molecular chain flow at the glass transition temperature of ≈115 °C, which enhances the van der Waals force for the printing process.^[^ ^51^ ^]^ This is indicated by the strong hysteresis effect in the approaching and withdrawing force–displacement curves (Figure 3d).^[^ ^52^, ^53^ ^]^ Additionally, the reduced height of the PS nanospheres at the glass transition temperature yields a larger van der Waals force and greater viscosity.^[^ ^32^, ^54^ ^]^

The necking of the PS nanospheres during the heating process is another factor in determining the transfer mechanism, as evidenced by the quantitative analysis of the adjoined region in the SEM image in Figure 3b. The necking extent of nanospheres gradually increases as the temperature grows from ambient temperature to 130 °C. In particular, at 130 °C, PS nanospheres undergo an irreversible fusing, ultimately leading to the disappearance of the particle‐to‐particle boundary. To determine the extent of necking, the contrast of SEM grayscale maps was acquired to manifest the electrical conductivity (Figure S12, Supporting Information). As temperature rises, the contrast of the adjoined nanosphere region in the grayscale map diminishes, suggesting enhanced necking. The AFM height result shows the height difference of the adjoining region of nanospheres is 121.1, 117.7, and 62.6 nm, respectively. Therefore, the enhanced necking between the nanospheres strengthens the inter‐particle binding force, resulting in increased printing efficiency, as presented in Figure 3f–h. Although the nanostructures themselves are not perfectly uniform in size, a conformal and high‐efficiency transfer can be achieved.

The present principle markedly diverges from the latest nanotransfer printing of planar nanostructures, which entails a printing temperature over 150 °C.^[^ ^26^ ^]^ By utilizing differential expansion and deformation between soft stamps and substrates due to heat‐induced changes, these methods rely on fracture at interfaces and resultant surface energy variations. In stark contrast, the proposed principle in this article is the decreased Young's modulus of PS nanospheres, self‐adhesive properties, and interparticle necking at the glass transition temperature. Our methodology contrasts previous printing techniques where the adhesion energy between nanostructures and the substrate persisted as a constant.^[^ ^55^ ^]^ This is further evidenced by the low printing efficiency of SiO_2_ with a similar diameter when subjected to identical printing temperatures (Figure S13, Supporting Information). When it comes to SiO_2_, due to the high melting point of silica materials,^[^ ^56^ ^]^ the temperature at which the planar interface is formed is much higher than that tolerated by PDMS soft stamp. Therefore, the efficient transfer of silica to a non‐adhesive layer is indeed a difficult challenge which needs further exploration.

### 2.3 Combined Thermal‐Assisted NP‐NTP and ITP for Multiscale Patterning

We have demonstrated metamolecule patterning consisting of colloidal nanospheres using thermal‐assisted NP‐NTP. In this section, the ITP technique is further introduced for deterministic control over macro patterns, which have been previously demonstrated to pattern quantum dots.^[^ ^57^ ^]^ Quantum dots (QDs) superlattice and patterning present a promising avenue for exploring novel physics, such as miniband effects,^[^ ^58^ ^]^ and for the realization of a variety of devices, including high‐resolution displays^[^ ^59^ ^]^ and photodetectors.^[^ ^60^ ^]^ However, the color of quantum dots originates from the light absorption determined by their bandgap characteristics. QDs of multiple materials and structures are typically required for a full‐color device. In contrast, the absorption of wavelength‐scale PS nanospheres in the visible range is very weak, with the color of PS nanosphere patterning originating from structural color. Furthermore, the patterning of wavelength‐scale PS nanostructures represents another significant scientific pursuit, potentially offering fresh insights into new physical effects, including nanoscale focusing effects^[^ ^61^ ^]^ and whispering‐gallery‐like modes.^[^ ^62^ ^]^ Moreover, the PS nanosphere patterns are quintessential and extensively utilized templates for fabricating other nanostructures.^[^ ^63^ ^]^

**Figure 4:**
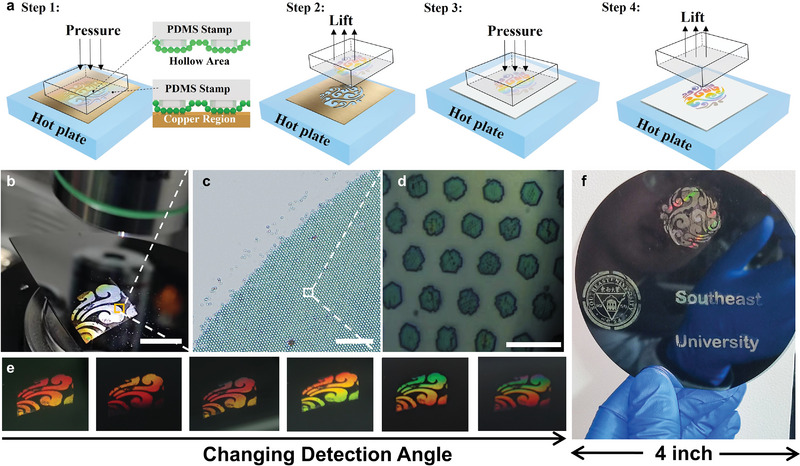
Combined thermal‐assisted NP‐NTP and ITP for multiscale patterning. a) Schematic diagram of the transferred macro pattern. Step 1: Print the PDMS on the copper intaglio under certain pressure at 115 °C. Step 2: After printing for 3 min, lift the PDMS up. Step 3: Print the PDMS on the wafer in the same condition. Step 4: Lift the PDMS up and finish the fabrication of multiscale pattern. b) The transferred buds at the macro scale. Scale bars, 1 cm. c,d) The optical images at different magnifications. Scale bars, c) 100, d) 10 µm, respectively. e) The visual appearance of the bud at different detection angles under an obliquely incident condition. f) The multiscale pattern was obtained on a 4‐inch silicon wafer consisting of a propitious cloud, a school badge, and the English letter “Southeast University”.

The proposed procedure, as illustrated in **Figure** **4**a, involves the transfer of closed‐packed PS nanospheres onto a PDMS stamp, which is then brought into contact with a copper intaglio. However, due to the limited contact area, ITP was also performed on the glass transition temperature of PS (115 °C) with a pressure of ≈5 N to facilitate the printing of PS nanospheres on the intaglio. The contacted part of the PS nanospheres with the copper intaglio was transferred to the copper, while the non‐contacted part of the PS nanospheres remained on the PDMS stamp, forming a deterministic macro scale patterning. This macroscale pattern, along with the micro pattern defined by the PDMS stamp, was further printed on the wafer using the thermal‐assisted NP‐NTP strategy. To further structural characterization from macro and micro aspects, we observed the constructed bud pattern under an optical microscope with different magnifications of objective. As shown in Figure 4b, a clear and holonomic macro pattern was visible, while the edges of the bud pattern were clearly visible under 5× objective (Figure 4c). The PS nanopatterns show a highly repetitive arrangement under 100× objective (Figure 4d), with a resolution of the pattern estimated through SEM or optical microscope images at ≈2757 PPI (Figure S14, Supporting Information). Thus, the combinatorial fabrication method of thermal‐assisted NP‐NTP and ITP provides a new strategy for constructing multiscale patterns with high uniformity, large‐scale, and high resolution. In the next section, we will show multiscale patterns of PS nanospheres with ≈2750 PPI is enough for the multi‐modal anti‐counterfeiting application. Furthermore, we recorded reflective images under oblique incident parallel light illumination with various detection angles to characterize the optical performance of the multiscale patterns (Figure 4e). The drastic changes in color spanning red, yellow, and green as the detection angle varies, indicating the presence of wavelength‐scale periodic structures in the micro‐region.^[^ ^64^, ^65^ ^]^ The sensitivity of the multiscale pattern to the detection angle confirms the long‐range ordering on the microstructure, the ability of spectrum splitting, and the potential of its anti‐counterfeiting applications.^[^ ^66^ ^]^

To demonstrate the large‐scale patterning ability and the potential to create arbitrary macroscale patterns, we created multiple macroscale features on a 4‐inch wafer, including multiscale patterns of a propitious cloud, school badge, and English letters of “Southeast University”. The specular image of the pattern showed almost a single color under omni‐direction illumination (Figure 4f), while the pattern showed brilliant color at oblique detection angles (Figure S15f,g, Supporting Information). It is important to note that the structure of the intaglio is crucial for creating specific patterns, and a full‐hollowed intaglio may not be suitable for patterning specific letters or characters. Therefore, semi‐hollowed copper intaglio was used for the words “Southeast University” (Figure S15a,b, Supporting Information). We also demonstrated the ability to create patterns of a Peking Opera facial makeup, a sunflower, bamboo forests, a sailboat, and the art Chinese character “Horse” (Figure S15c–e, Supporting Information). For full‐hollowed copper intaglio, a relatively larger pressure in the first step can be used to remove the contact part completely. However, in the case of semi‐hollowed copper, the pressure applied in the first step should be carefully chosen to avoid unexpected contact between the deformed PDMS stamp and the semi‐hollowed region. By marrying ITP with thermal‐assisted NP‐NTP, we could define nanosphere patterns with hierarchical length scale control, arranging nonplanar nanostructures with patterns up to a 4‐inch scale.

### 2.4 Multi‐Modal Anti‐Counterfeiting with Multiscale PS Nanosphere Pattern

The multiscale pattern with unique optical reflections holds promise for various applications, including wearable sensors, flexible displays, and anti‐counterfeiting patterns.^[^ ^5^ ^]^ The fundamental idea is to utilize the functionality of nano and micro scales to generate emergent behaviors at the macro scale.^[^ ^67^ ^]^ In this section, we introduce the concept of multi‐modal anti‐counterfeiting using our multiscale patterns. The idea behind multi‐modal anti‐counterfeiting is based on the macro‐optical response of multiscale patterns under different light excitations.

**Figure 5:**
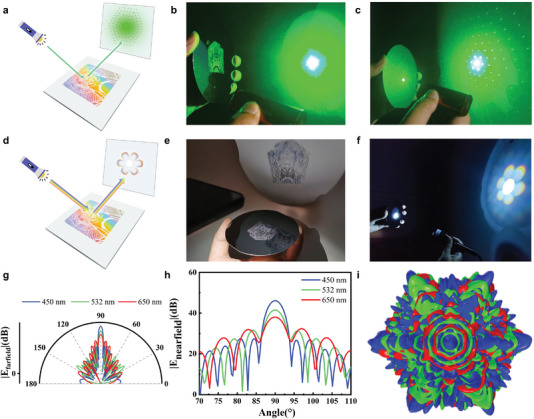
Concept of multi‐modal anti‐counterfeiting. a) Schematic diagram of laser anti‐counterfeiting mode. b) Reflective pattern with the green laser irradiating the blank area in the wafer. c) Diffraction patterns with the multiscale patterns under the excitation of green laser. d) Schematic diagram of collimated white light anti‐counterfeiting mode. e) Mirror image of “Peking Opera facial makeup” pattern under omnidirectional illumination. f) The “Peking Opera facial makeup” pattern became “seven‐colored flowers” under the collimated white light excitation. g,h) The far‐field simulation results at different detection angles under excitation of three wavelengths, respectively. i) 3D far‐field superposition scattering pattern with three excitations of different wavelengths.

Under laser excitation, the long‐range‐ordered micro hexagonal dense arranged nanostructure can create macro scale light diffraction patterns.^[^ ^13^ ^]^ In our laser anti‐counterfeiting mode, we use the reflective diffraction patterns of multiscale PS nanosphere patterns under laser excitation (as shown in **Figure** **5**a; Figure S16, Supporting Information). The 532 nm laser with a beam diameter of 2 mm was used to excite the sample, and a white screen as the receiving screen. When the green laser shines on a location on the wafer without structure, the laser is reflected directly, forming a bright spot on the screen (as shown in Figure 5b). However, when the green laser is directed at the pattern with nanoarrays, a well‐ordered and regular hexagonal reflective diffraction pattern is formed, with the brightest spot at the center and the reflection intensity of the spot gradually decreasing from the center to the surroundings (as shown in Figure 5c). In general, colloidal nanostructures are difficult to achieve ideal diffraction patterns using self‐assembly methods due to the randomness in their assembly process, which can cause blurring of diffraction patterns due to multi‐layers, defects, and grain boundaries at different scales.^[^ ^13^ ^]^ Although the nanoscale arrangement still has slight defects, it does not cause blurring of the diffraction pattern.^[^ ^68^ ^]^ Additionally, we performed a diffraction pattern experiment under a 450 nm laser, and we obtained similar imaging results. We obtained uniform diffraction patterns for random positions of the multiscale patterns (as shown in Movie S2, Supporting Information), demonstrating the outstanding consistency of the structure over a large area. Notably, the spatial location of the diffraction pattern of blue light is different from that of green light, indicating the spectrum‐splitting properties at the centimeter scale (Figure S17 and Movie S3, Supporting Information).

Building on the spectrum splitting phenomenon, we propose the concept of anti‐counterfeiting under white light excitation mode (schematic is shown in Figure 5d). When omnidirectional illumination was used for irradiation, the receiving screen displays a mirror image of the macro pattern (Figure 5e; Figure S16e, Supporting Information). However, when quasi‐parallel light from a broad‐spectrum white tungsten‐halogen light source with a collimator was used for excitation, the reflection pattern of “Peking Opera facial makeup” transforms into “seven‐colored flowers” (Figure 5f; Movie S4, Supporting Information). Specifically, the Seven‐color flowers have bright white cores and hexagonally distributed petals with colored edges. Additionally, multiple petals surround the hexagonal petals, and the intensity of the outer petals progressively darkens, consistent with the diffraction patterns under the laser excitation. We further show the multi‐modal anti‐counterfeiting patterns under two white light sources simultaneously in Figure S18 (Supporting Information).

To explore the mechanism of multi‐modal anti‐counterfeiting, we perform the 3D finite element method (FEM) for modeling. The modeling details are in Experimental Section and Figure S19 (Supporting Information). The simulation uses planar electromagnetic waves with wavelengths of 450, 532, and 650 nm (as representatives of RGB color) as excitation light sources. Periodic hexagonal‐arranged PS metamolecules (with 52 hexagonal‐arranged PS nanospheres) are placed upon the silicon substrate, similar to the architecture in our experiment. For all wavelengths of the incident light, the 2D far‐field scattered intensity maps (Figure 5g) and 3D far‐field scattered intensity maps (Figure S19d, Supporting Information) have multiple side lobes at different angles, illustrating the cause of the diffraction pattern at the macroscopic scale. The scattering intensity of decay from the center of the 90° detection angle to both sides, explaining the gradual weakening of the laser diffraction spot in Figure 5b. Nearfield simulation (Figure S19c, Supporting Information) further illustrates the wave vector directions of the different diffraction levels, indicating the origin of the macro diffraction patterns. Additionally, the far‐field scattering results show significant variations in the amplitudes of the three RGB electromagnetic waves for different detection angles, confirming that the spatial location of the diffraction pattern varies for different wavelengths of laser excitation (Figure 5h).

Furthermore, we have explained the cause of the seven‐color flower pattern under the excitation of quasi‐parallel white light. Due to the different coupling abilities of electromagnetic waves with various frequencies, electromagnetic waves of different wavelengths are enhanced or suppressed, resulting in spectrum splitting and the resultant coloring performance.^[^ ^26^ ^]^ Therefore, the reflection displays a span of rainbow color on a macro scale. Additionally, the scattered intensity distribution at the angle near the specular reflection (90°) reveals that the scattered lights of blue, green, and red colors are not entirely separated in space. Under quasi‐parallel white excitation, scattering of blue, green, and red light simultaneously exists at various angles, resulting in some regions of the flower pattern appearing white (as shown in Figure 5f). Finally, we recolored and superimposed the calculated 3D far‐field scattering pattern (as shown in Figure S19d, Supporting Information) with red, green, and blue colors for different wavelengths of excitation on the same map to generate the flower‐like pattern (as shown in Figure 5i), which matches well with the far‐field anti‐counterfeit pattern under the quasi‐parallel white light excitation.

Compared to recent multi‐pattern anti‐counterfeiting techniques, our approach is based on a distinct principle. Our fabrication process is cost‐effective, and the anti‐counterfeiting detection method is more user‐friendly. For instance, multi‐pattern anti‐counterfeiting techniques based on metasurface, fabricated using electron beam lithography, are limited by the complexity of the process and submillimeter scale.^[^ ^69^ ^]^ A complex optical path is required to project the patterns, resulting in the excitation of small areas. In contrast, our proposed method stores multiple pattern information in an inch‐scale pattern, requiring only a simple laser pointer or macro‐scale quasi‐parallel light to excite it. Furthermore, multiple patterns can be generated using materials with different luminescence and lifetimes combined with time‐gating technology.^[^ ^70^ ^]^ In contrast, our approach uses a single material, requiring less material synthesis, excitation, and detection.

**Figure 6:**
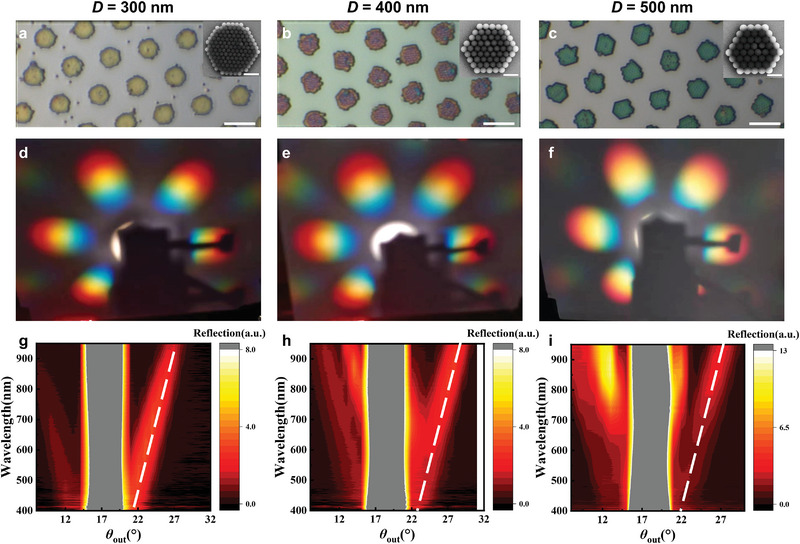
The tunable optical and anti‐counterfeiting properties of the multiscale pattern. a–c) The bright‐field micro‐region optical images of multiscale patterns with a) 300 nm, b) 400 nm, and c) 500 nm diameter nanospheres. Scale bar, 5 µm. The insets show the SEM image of a single metamolecule. Scale bar, 1 µm. d–f) The experimental diffraction pattern of d) 300, e) 400, and f) 500 nm diameter PS nanospheres under the excitation of collimated white light. g–i) The angle‐resolved spectra of the multiscale patterns with a) 300, b) 400, and c) 500 nm diameter PS nanospheres under the excitation of collimated white light. In all angle‐resolved spectra, the specular reflection is located at 18° while the first‐order diffraction ranges from 22° to 25°.

We further demonstrated the tunable optical and anti‐counterfeiting properties of the multiscale pattern by adjusting the pitch of the periodic metamolecules (i.e., the center‐to‐center distance between adjacent metamolecules), nanosphere diameter, and the number of nanospheres in one metamolecule (periodic element). The simulation results show that by changing the pitch of the periodic metamolecules, the spatial distribution profile of the far‐field diffraction pattern is significantly altered. The number of diffraction orders in the petal‐like pattern is reduced when the pitch is smaller (Figure S20, Supporting Information). By contrast, when changing the size and number of nanospheres, the spatial distribution profile of the far‐field diffraction pattern remains similar. The angle distribution of different diffraction orders is essentially the same, with only differences in relative intensity (Figures S21 and S22, Supporting Information). Experimentally, we fabricated multiscale patterns from 300 nm to 500 nm diameter nanospheres and compared their microscopic and macroscopic optical properties. It can be observed from **Figure** **6**a–c that the micro‐region reflection images of the metamolecules show distinctive colors as yellow (300 nm), red (400 nm), and green (500 nm). The micro‐region spectra from the microscope and the reflection spectra from the macroscale integrating sphere both show a red shift dip with the increase of nanosphere diameter, yielding different mirror image colors under white light omnidirectional illumination (Figure S23, Supporting Information). We also built a homemade goniometric set‐up to characterize the macroscale diffraction pattern and angle‐resolved spectra under the collimated white light excitation (Figure S24, Supporting Information). As shown in Figure 6d–f, the profiles of diffraction patterns are almost the same in angle distribution with slight intensity variation for the same color. This phenomenon is further verified by the angle‐resolved spectra. (Figure 6g–i) Under the excitation of a collimated white light incident at 18°, except for specular reflection, the diffraction shows a similar angle distribution with a reflection maximum from 22° to 25° across the visible range. In addition, we noticed that for smaller nanospheres (300 nm diameter), the diffraction of blue light is stronger, while the larger nanospheres (500 nm diameter) show a red‐shift maximum reflection wavelength. In this regard, we demonstrated a paradigm of imparting nanoscale, microscale, and macroscale patterning structures into macrofunctional devices.

## 3 Conclusion

In this study, we accomplished multiscale nanostructure patterning by thermal‐assisted nonplanar NP‐NTP technology. We achieved long‐range order transfer printing with an impressive efficiency of up to 97.2% within minutes. Additionally, by combining intaglio TP technology, we could pattern multiscale designs on a 4‐inch wafer, achieving a resolution of over 2750 PPI.

Although the proposed thermal‐assisted transfer printing method has greatly improved the long‐range order of multiscale patterns, there are still some directions worth exploring in the future. For the assembly process, slight perturbations at the solution interface may affect the uniformity and integrity of the membrane. In situ detection techniques, such as optical microscopy, need to be introduced for precise monitoring and control of assembly and transfer printing processes. As for the transfer printing process, since PDMS is a flexible template, it will deform when subjected to pressure, resulting in an uneven force on the substrate. Introducing a roller tool to make the localized force uniform and improve the long‐range order of the multiscale patterns is also recommended.^[^ ^26^ ^]^ In addition, due to the melting point lowering effect of nanomaterials,^[^ ^56^ ^]^ we believe that such a nanotransfer printing technique harbors the potential for extension to diverse nanomaterial systems, including polymer nanostructures, plasmonic nanostructures, and semiconductor nanocrystals.

We also introduced the concept of multi‐modal anti‐counterfeiting based on the multiscale patterns, which can be activated by different light signals to produce various anti‐counterfeiting optical patterns at both macro and micro scales. We clearly illustrate that under the laser anti‐counterfeiting mode and collimated white light excitation anti‐counterfeiting mode, the spatial distribution profile of the diffraction pattern is determined by the pitch of the metamolecules, while the number and diameter of the nanospheres can adjust the intensity. Under the omnidirectional illumination white light illumination mode, the inch‐scale intaglio pattern determines the pattern of the mirror image, whereas the nanoscale structure determines the color of the mirror image. Holographic patterns will be introduced in future endeavors using Fourier transform methodologies.^[^ ^71^ ^]^ Incorporating nanoscale absorption structures such as plasmonic structures makes it feasible to realize ultra‐thin structural color devices with unusual appearances and superior color purity.^[^ ^72^ ^]^ Consequently, the low cost of colloidal nanostructures can be harnessed to achieve large‐scale, high‐throughput processing and commercialization of structural color optical display devices. We believe this innovative multiscale NP‐NTP strategy will open up new possibilities for bridging the functionality of micro and nanostructures into transformative devices.

## 4 Experimental Section

### Materials

Polystyrene (PS) nanospheres (Size: 300 nm, 400 nm, and 500 nm, Homogeneity CV%: 3%) were purchased from Huge Biotechnology Company (Shanghai, China). Sodium dodecyl sulfate (SDS) was purchased from Sigma‐Aldrich (St. Louis, Missouri, USA), and hexane was purchased from Kelon Chemical (Chengdu, China). The resistivity of the ultrapure water used in the experiment was >18.2 MΩ·cm (Milli Q). All chemicals were used without any purification. A 6‐inch PDMS stamp is commercially available.

### Interfacial Assembly of PS Nanospheres

The PS nanospheres were assembled in a 100 mL crystallizing dish. Initially, SDS aqueous solution (2 wt.%,100 µL) was injected into 40 mL ultra‐pure water to lower the surface tension of the water. Next, hexane (4 mL) was applied to the surface, and the hexane‐water interface was formed naturally. Subsequently, several drops of PS nanosphere colloid (2.5 wt.%, dispersed in ethanol‐water mixed solution with a volume ratio of 1:1) were added from the edge of the crystallizing dish slowly (3 s each drop) till the complete formation of the monolayer at the interface. After the hexane evaporated (≈10 min), the closely packed PS nanospheres were transferred to a silicon wafer for further characterization. A syringe was used to refill the water several times to remove PS nanospheres dispersed in the aqueous phase. It should be noted to avoid damage to the film in this process.

### The Transfer Printing of PS Nanosphere Films

Before transferring PS nanosphere films, the PDMS stamp was treated with oxygen plasma (Harrick, Pleasantville, USA) with a power of 50 mW for 2 min to increase surface free energy and hydrophilic property. Subsequently, SDS aqueous solution (≈10 µl) was added to create a gap in the film for the PDMS stamp to probe into the water. Next, the PS nanosphere monolayer was transferred to the patterned PDMS stamp via the LB transfer technique.^[^ ^38^ ^]^ After transferring, let the substrate dry naturally without disturbance, preventing the nanospheres from dislocation. Before printing, the silicon substrates were also oxygen plasma treated to increase surface free energy. The printing process was conducted on a heating plate at the temperature of 100, 110, 115, and 130 °C, respectively. In a typical printing process, the PDMS stamps were applied onto the silicon substrate with a uniform force of 5 N for 1–15 min.

### Combined Thermally Assisted NP‐NTP and ITP for Multiscale Patterning

The PS NP film on the PDMS was prepared according to work introduced above. Silicon wafer and copper substrate with a hollowed pattern were preheated to 115 °C on a heating plate. Apply the structured surface of PDMS to the copper intaglio (also treated by oxygen plasma). A specific pressing force (≈20 N, 10 s) was exerted to remove redundant parts of the film, retaining the macro pattern. The PDMS was then applied to the silicon wafer with uniform pressure (≈5 N, 3 min). Nanostructures with both macro and micro patterns were thus printed on the silicon wafer for further characterization.

### Optical and Structural Characterization

The morphological and structural studies were carried out using scanning electron microscopy (SEM, Zeiss Ultra Plus, German) at an accelerating voltage of 5.0–20.0 kV. ImageJ software was used to conduct FFT of the SEM images and the analysis of the printing efficiency. Micro‐region optical photographs were taken from an inverted microscope (Nikon Ti‐U). A 100×/0.80 NA objective lens (Nikon Plan Fluor ELWD 100×) was used to collect micro‐region reflection images and spectra, and the micro‐region spectra were recorded by a spectrographic CCD (Acton SpectraPro SP2550i equipped with Pix‐256E camera, Princeton Instruments). All spectra were corrected by subtracting the CCD's dark current and normalized as a reference spectrum, which was recorded in an area without particles. The macroscopic reflection spectrum was taken from a fiber optic spectrometer (Nova, Ideaoptics Co., Ltd., Shanghai, China) and an integrating sphere (IS‐30‐6‐R). Metallographic microscope with 10× and 100× objective lenses (Angwei, ShangHai) were used to take the optical images of the patterned nanostructure. The MATLAB program was used to perform grayscale transformation and static analysis on SEM images.

### AFM Mechanical Characterization and Height Characterization of PS Nanosphere

The Young's modulus and height characterization of PS nanosphere was conducted with an Atomic Force Microscopy (AFM, Ntegra Prima, NT‐MDT Spectrum Instruments, Russia) analysis. The spring constants of the cantilevers were determined through calibration of the AFM probe (Tap150GB‐G, NSG30) on a hard Al_2_O_3_ substrate (Figure S25, Supporting Information), cross‐checked with fundamental resonant frequency calibration. The temperature‐variable AFM tip indentation was performed using probes, and force–displacement curves were measured during approaching and withdrawing. Analysis software utilizing Hertz's model for contact mechanics was employed to fit force–displacement approaching curves at various temperatures (85, 100, 110, 115, 130 °C) after ≈10 min heating, with an AFM tip indentation being used to calculate the Young's modulus.

### Muti‐Modal Optical Anti‐Counterfeiting of Multiscale Patterned PS Nanospheres

In Figure 5b,c, a dual‐color laser pointer was used to illuminate the multiscale pattern samples. In Figure 5e,f, an omnidirectional LED and a power‐tunable Nikon halogen lamp with a collimating fiber were respectively used to illuminate the multiscale patterned samples. The substrate with PS pattern and the screen were placed in parallel with a small angle to collect the reflective diffraction pattern.

### Optical Simulation of Multiscale PS Nanosphere Pattern

3D FEM electromagnetic simulation was conducted with commercial software (COMSOL Multiphysics). As the periodic unit structure, a regular hexagonal prism was extruded by a hexagonal working plane, with a hexagonal edge length set to 4.04 µm (Same to the structure of the unit structure of the PDMS stamp). The regular hexagonal prism is divided into several spatial areas. The perfect matching layers at the top and the bottom prevent light reflection at the boundary. Layer sandwiched in the middle was the air layer, the PS metamolecule layer (52 nanospheres with a diameter of 500 nm), and a silicon layer (Figure S6, Supporting Information). The refractive index of air was set to 1, and the refractive index of silicon and PS spheres was derived from the optical handbook. Periodic boundary conditions are used on the six sides of the regular hexagonal prism to simulate the optical properties of the infinite periodic structure. A working plane is inserted into the air layer as a port to provide plane wave excitation that propagates along the −*Z* direction and polarizes in the *y* direction. The air layer area above the port was set as the far field area to calculate the angular distribution of the far field scattered light intensity. In the entire simulation area, the grid size was set to 20–100 nm (Figure S19b, Supporting Information). The 2D far‐field scattering pattern was calculated at the *y–z* plane indicated in Figure S19e (Supporting Information).

## Conflict of Interest

The authors declare no conflict of interest.

## Author Contributions

D.S., W.‐L.W., P.‐Q.S., and Y.‐C.Y. contributed equally to this work. D.S., W.‐L.W., and P.‐Q.S. developed overall methodology and carried out the theoretical analysis of NP‐NTP. W.‐L.W., and P.‐Q.S. conducted the assembly and printing experiment. D.S., Z.X.C., and Y.F.Z. performed the optical characterization, K.Y.B. and H.‐L.Z. performed the anti‐counterfeiting experiments. Y.‐C.Y. developed the 3D optical modeling and simulation analysis. All authors contributed to the manuscript preparation and revision. T.Z. supervised the work team.

## Supporting information

Supporting Information

Supplemental Video 1

Supplemental Video 2

Supplemental Video 3

Supplemental Video 4

## Data Availability

The data that support the findings of this study are available in the supplementary material of this article.
