# Supplementary material for: Thermal‐Assisted Multiscale Patterning of Nonplanar Colloidal Nanostructures for Multi‐Modal Anti‐Counterfeiting
Source: Adv Sci (Weinh). 2023 Oct 22;11(1):2305469. doi: 10.1002/advs.202305469 (PMC10767423; doi:10.1002/advs.202305469)
Supplement: Supplementary file 1 — Supporting Information [file ADVS-11-2305469-s001.pdf]

ADVANCED SCIENCE
Open Access

Supporting Information

for *Adv. Sci.*, DOI 10.1002/advs.202305469

Thermal-Assisted Multiscale Patterning of Nonplanar Colloidal Nanostructures for Multi-Modal Anti-Counterfeiting

*Dan Su, Wei-Long Wu, Pan-Qin Sun, Yu-Chen Yuan, Ze-Xian Chen, Yun-Feng Zhu, Kai-Yu Bi, Huan-Li Zhou and Tong Zhang**

Supplementary Information

for

Thermal-assisted Multiscale Patterning of Nonplanar 3D Colloidal Nanostructures for Multi-mode Anti-counterfeiting

Dan Su[1,2,3]†, Wei-Long Wu[1]†, Pan-Qin Sun[1]†, Yu-Chen Yuan[1]†, Ze-Xian Chen[1], Yun-Feng Zhu[1], Kai-Yu Bi[3, 4], Huan-Li Zhou[1], Tong Zhang[1,2,3]*

[1]Joint International Research Laboratory of Information Display and Visualization, School of Electronic Science and Engineering, Southeast University, Nanjing 210096, China

[2]Key Laboratory of Micro-Inertial Instrument and Advanced Navigation Technology, Ministry of Education, School of Instrument Science and Engineering, Southeast University, Nanjing 210096, China

[3]Suzhou Key Laboratory of Metal Nano-Optoelectronic Technology, Southeast University Suzhou Campus, Suzhou 215123, China

[4]College of Software Engineering, Southeast University, Nanjing, Jiangsu 210096, China

*Corresponding author. Email: tzhang@sen.edu.cn (T.Z.)

†These authors contributed equally to this work.

Supplementary Figure 1-25

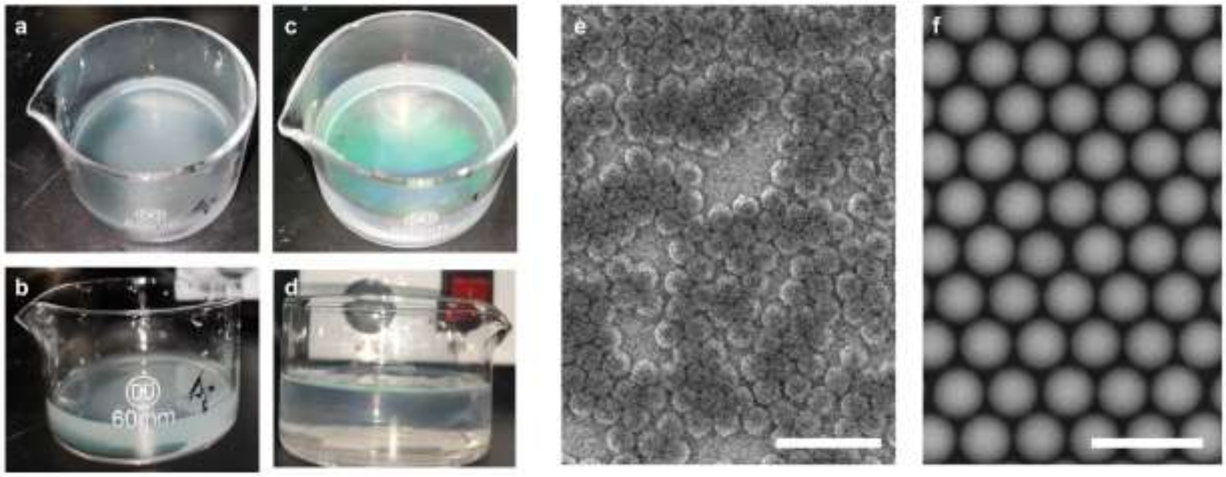

Supplementary Fig. 1 The hexane and SDS induced interfacial assembly of PS nanospheres. The solution image without (a-b) or with (c-d) hexane in the assembly system of PS nanofilms. The SEM image without (e) or with (f) SDS in the assembly system of PS nanofilms. Scale bar, 2 μm.

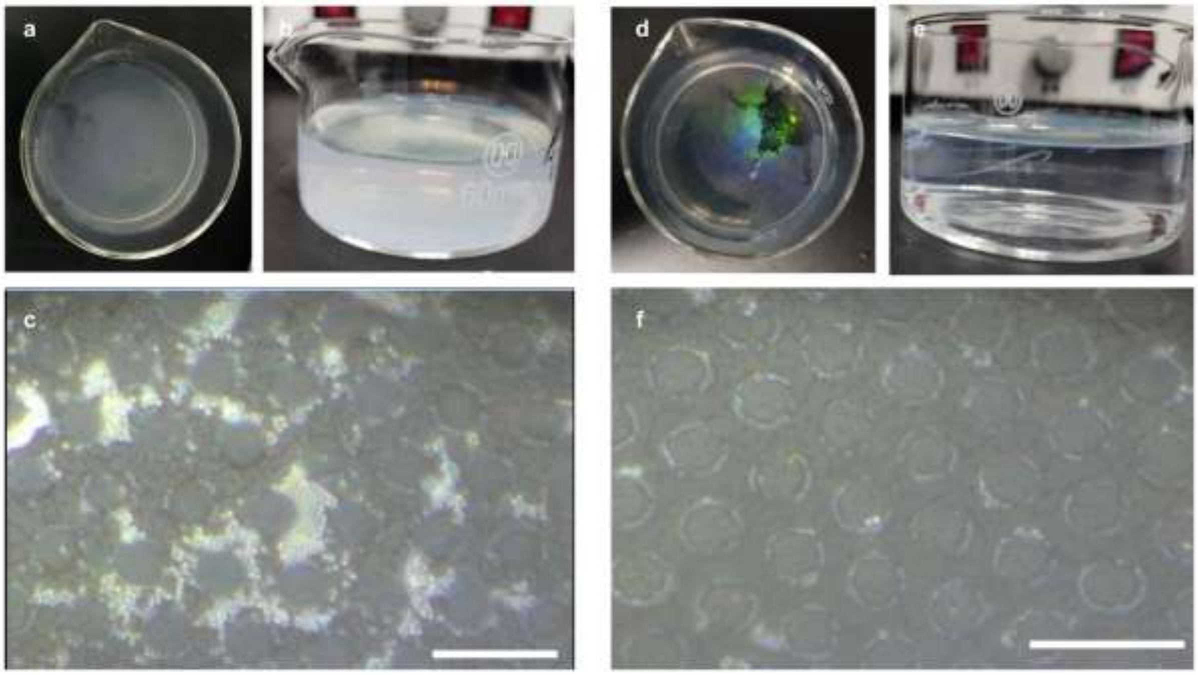

Supplementary Fig. 2. The impact of the refilling process on the quality of film obtained by the LB transfer method. a-b The nanofilm assembled at the interface without refilling process shot from the top (a) and lateral (b) sides of the container, respectively. **c** Corresponding optical microscopic images of the nanopatterns on the PDMS. Scale bar, 10 μm. **d-e** The film was assembled at the interface with a refilling process shot from the top (d) and lateral (e) sides of the container, respectively. **f** Corresponding optical microscopic images of the nanopatterns on the PDMS. Scale bar, 10 μm.

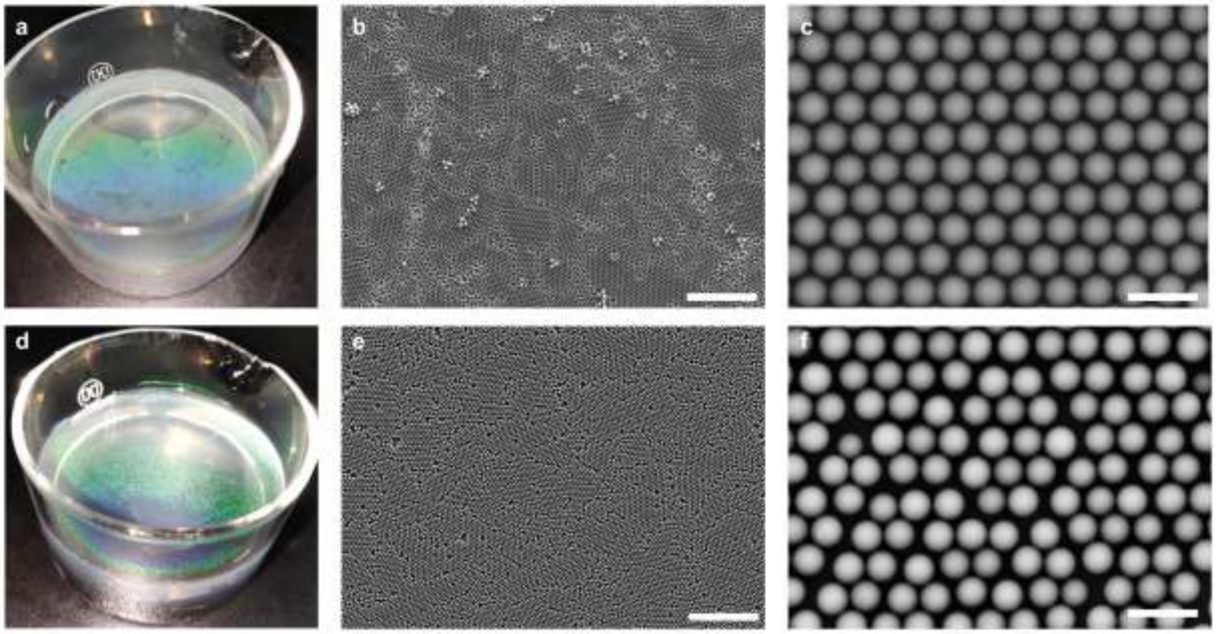

Supplementary Fig. 3. Assembly of PS and SiO_2 nanospheres. a Photo of nanofilm of PS nanosphere assembled at the interface. **b-c** SEM image of PS nanofilm transferred directly onto a

silicon substrate. Scale bar, 10 μm, and 2 μm, respectively. **d** Photo of nanofilm of SiO_2 nanosphere assembled at the interface. **e-f** SEM image of SiO_2 nanofilm transferred directly onto a silicon substrate. Scale bar, 10 μm, and 2 μm, respectively.

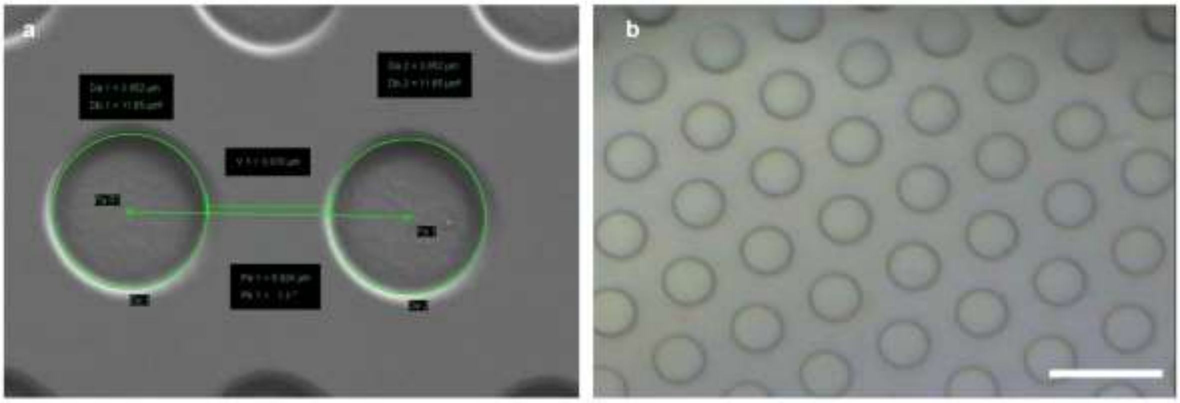

Supplementary Fig. 4. Characterization of the PDMS stamp. a SEM image of the Si hard template for duplicating PDMS stamp. **b** Optical microscopy image of the surface of PDMS stamp. Scale bar, 10 μm.

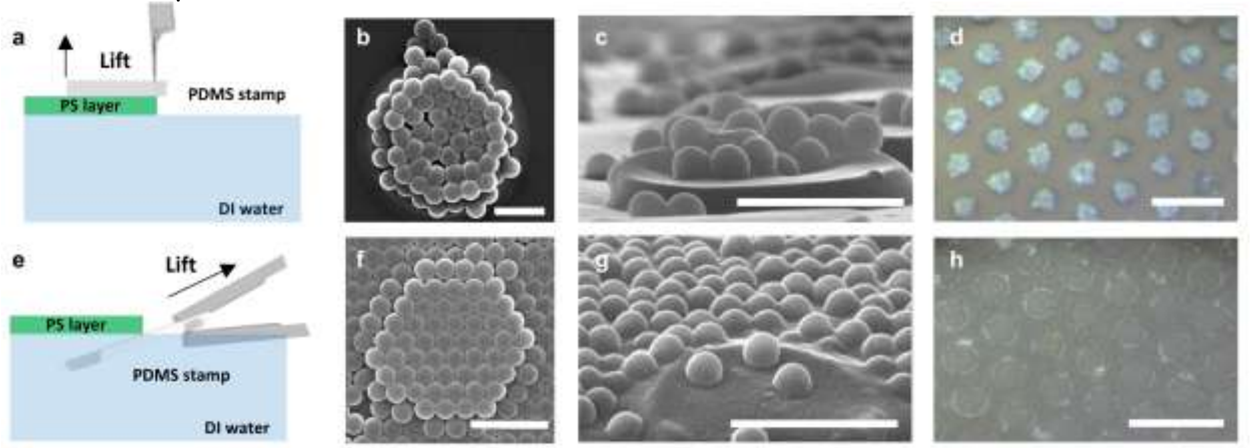

Supplementary Fig. 5. Nanostructure transferred with LS and LB transfer methods. a Schematic diagram of LS transfer method. **b** SEM image of individual nanopattern transferred by LS transfer method. Scale bar, 2 μm. **c** Nanopattern on PDMS using LS transfer method. Scale bar, 2 μm. **d** Optical microscopic image of nanopatterns on the PDMS transferred by LS transfer method. Scale bar, 10 μm. **e** Schematic diagram of LB transfer method. **f** SEM image of individual nanopattern transferred by LB transfer method. Scale bar, 2 μm. **g** Nanopattern on PDMS using LB transfer method. Scale bar, 2 μm. **h** Optical microscopic image of nanoarrays transferred by LB transfer method. Scale bar, 10 μm.

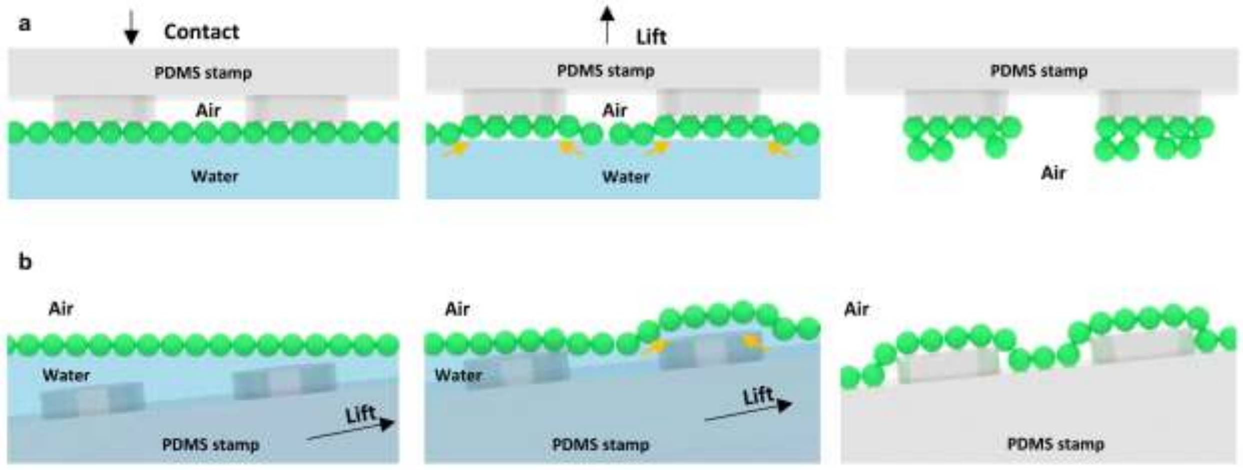

Supplementary Fig. 6. Schematic diagram of the LB transfer method and the LS transfer method. a The PDMS-Air-PS layers formed during the LS transfer method. **b** The PDMS-water-PS layers formed during the LB transfer method.

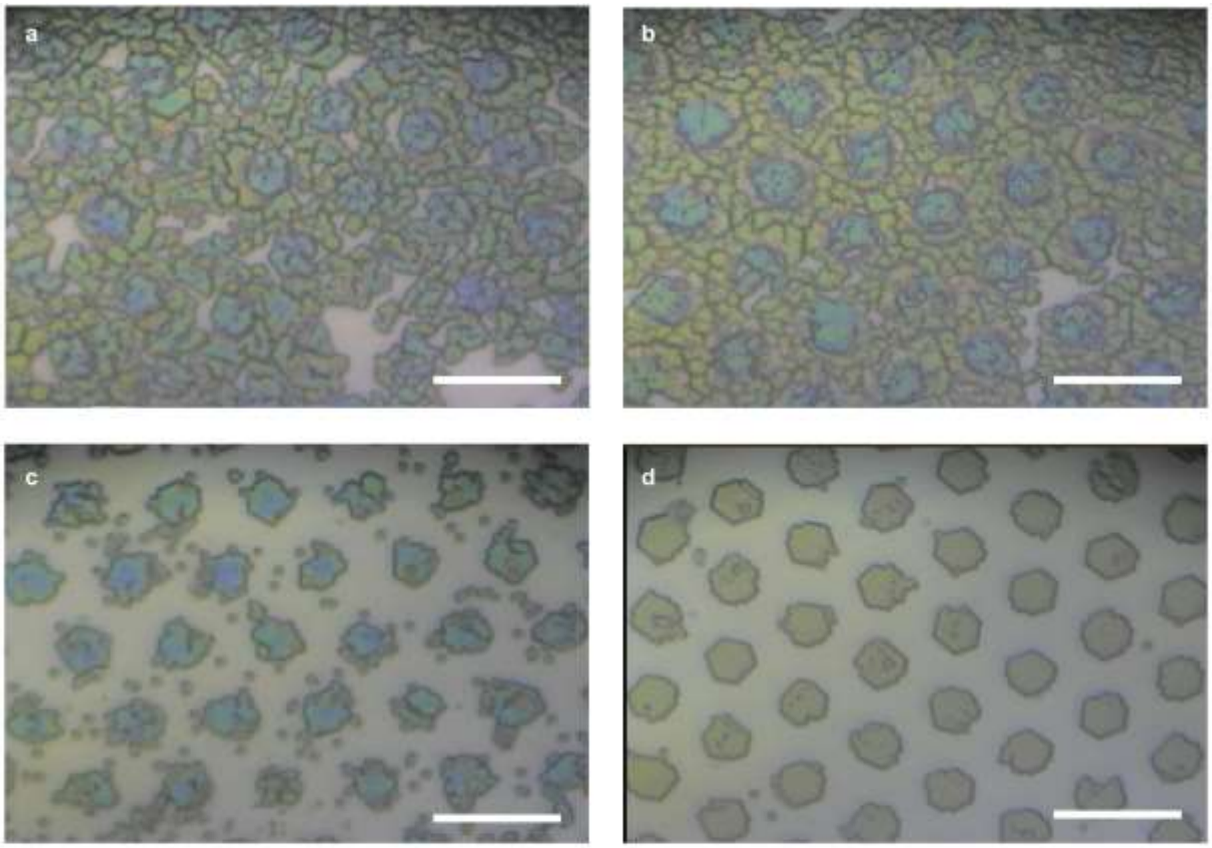

Supplementary Fig. 7. Pressure-dependent transfer printing results. a-d Nanopattern printed at different pressures for 1 min under optical microscopy observation, 25 N, 12 N, 10 N, and 5 N, respectively. Scale bar, 10 μm.

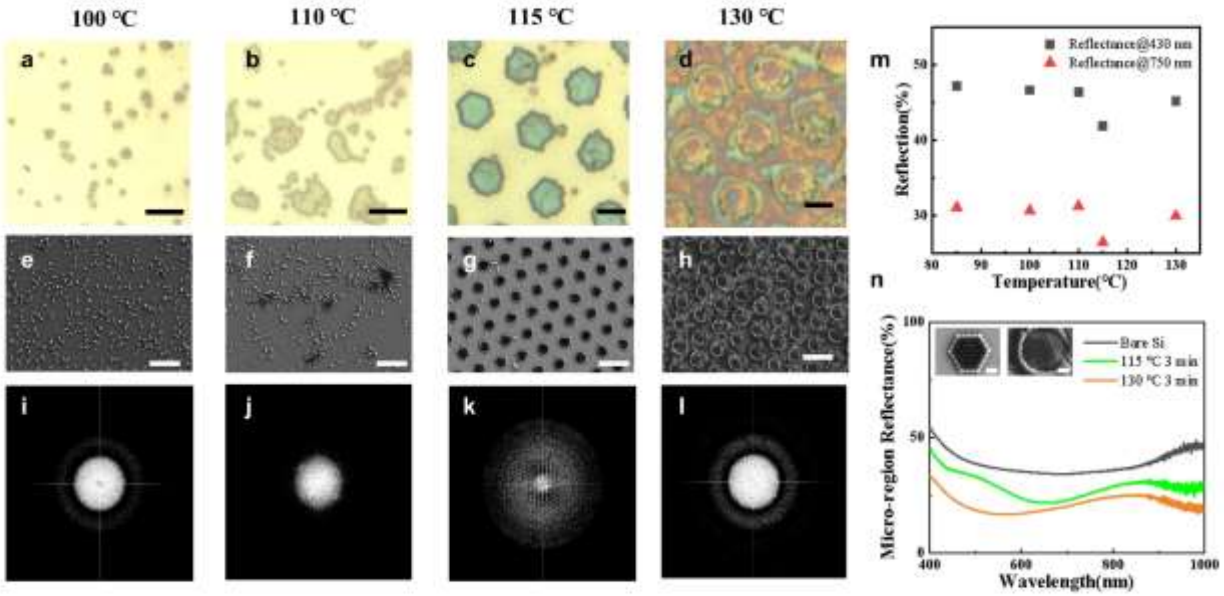

Supplementary Fig. 8. Effect of temperature on transfer printing. a-d Optical microscopic images of the nanopatterns printed at different printing temperatures. Scale bar, 2 µm. **e-h** Corresponding SEM images of the nanopatterns printed at different printing temperatures. Scale bar, 10 µm. **i-m** Corresponding FFT images of the nanopattern. **n** Comparison of reflectance of the nanopatterns printed at different temperatures at 430 nm and 750 nm. **o** Reflectance spectra of the PS metamolecule nanopatterns printed at different temperatures.

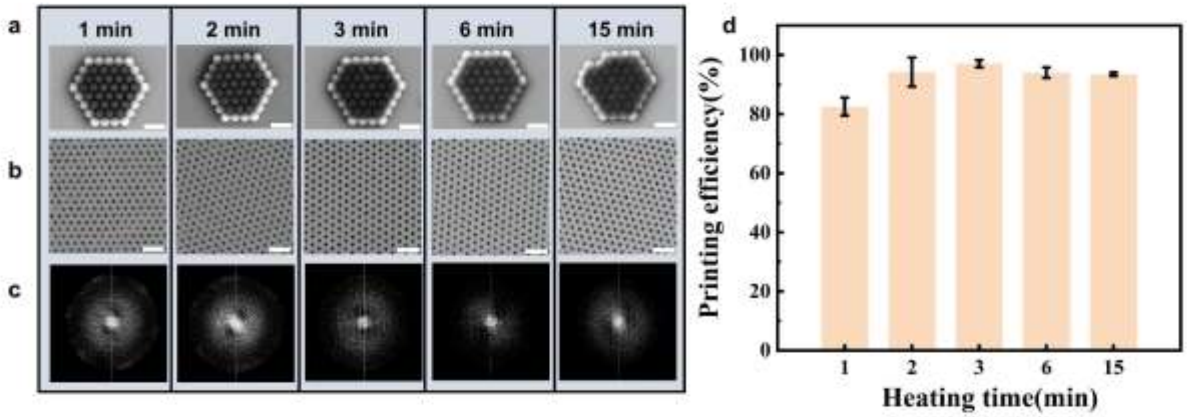

Supplementary Fig. 9. Multiscale PS nanosphere patterns under different heating duration at a printing temperature of 115°C. a-b SEM images of PS nanosphere assembly heated under 115°C for 1, 2, 3, 6, and 15 min (from left to right). Scale bar, 1 µm, and 20 µm, respectively. **c** FFT image of PS nanosphere assembly heated for a different duration. **d** Printing efficiency of PS nanosphere films on silicon wafers with different heating duration at 115°C.

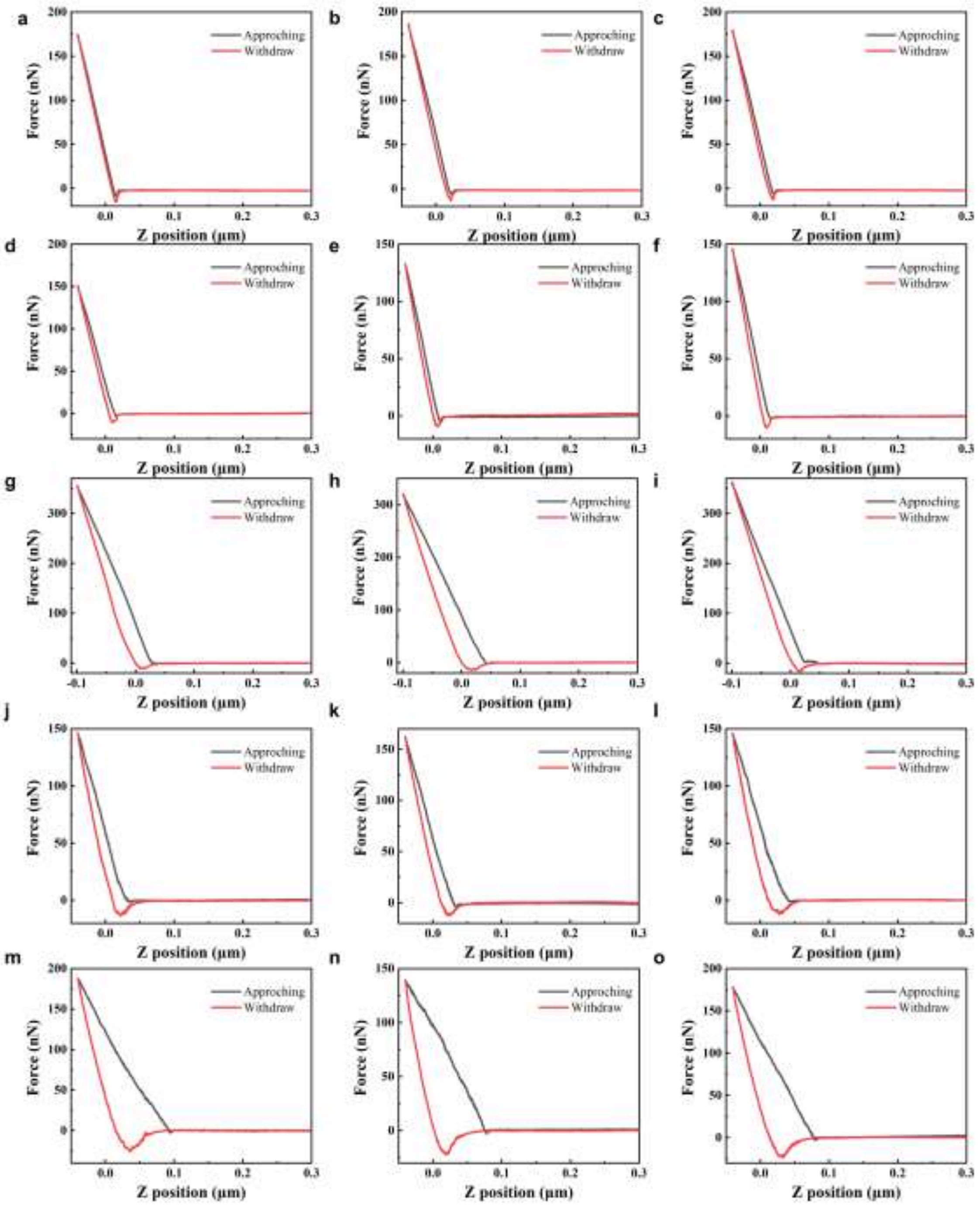

Supplementary Fig. 10. Temperature-dependent force curves for the calculation of Young's modulus. a-c Measured at 25℃. **d-f** Measured at 100℃. **g-i** Measured at 110℃. **j-l** Measured at 110℃. **m-o** Measured at 130℃.

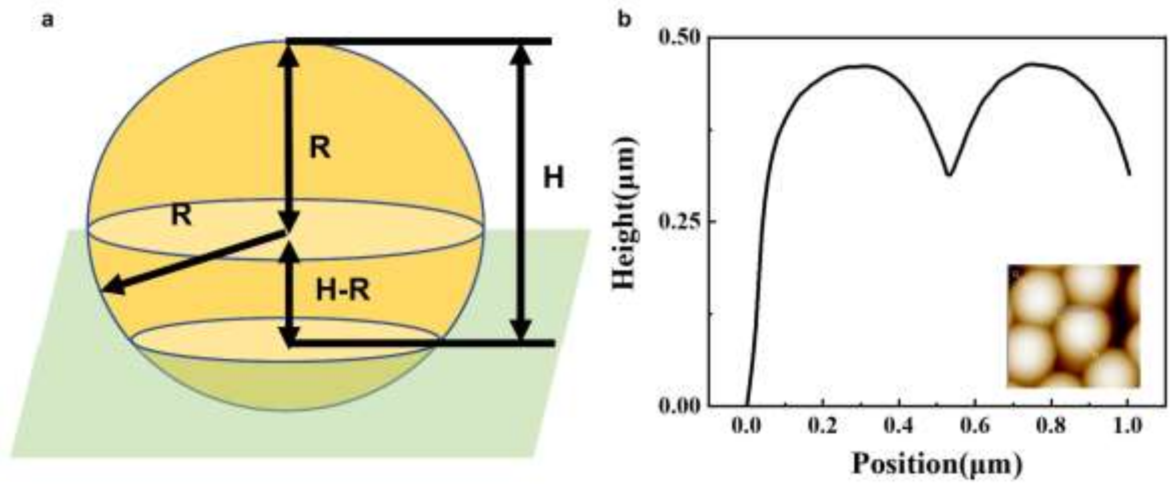

Supplementary Fig. 11. Spherical approximation to calculate contact area. a Schematic diagram of the spherical approximation. **b** Height diagram of nanosphere at room temperature.

The contact area of nanospheres and substrate is calculated as follows:

$$S = \pi[R^2 - (H - R)^2]$$

where R is the radius of the nanosphere at room temperature, H is the height of the nanosphere as read from the height map.

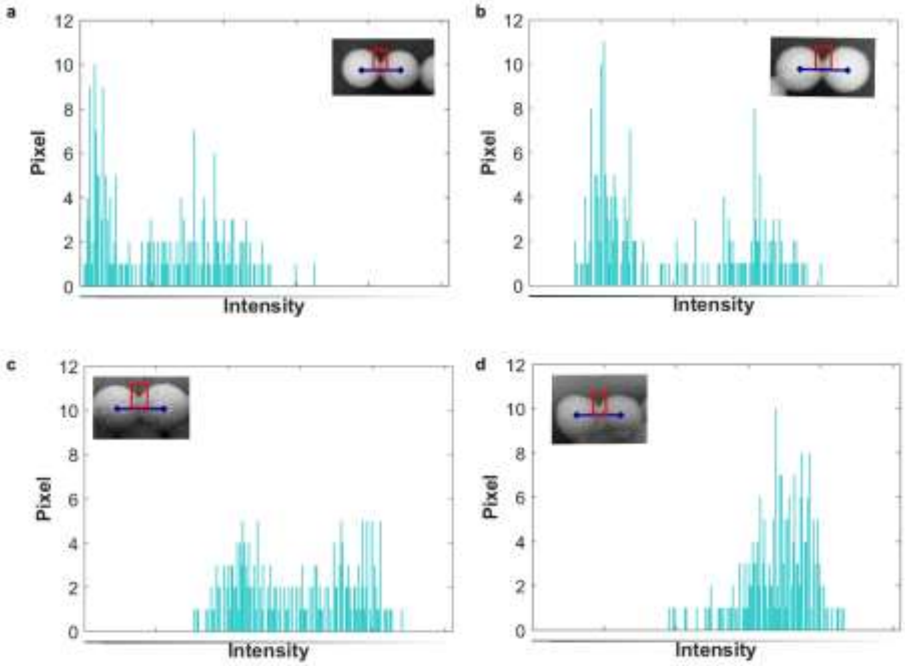

Supplementary Fig. 12. The grayscale analysis for the nanosphere necking regions under different printing temperatures. a-d The distribution of MATLAB grayscale calculations for the

selected junction region in the red rectangle at the printing temperature of 100℃, 110℃, 115℃, and 130℃, respectively.

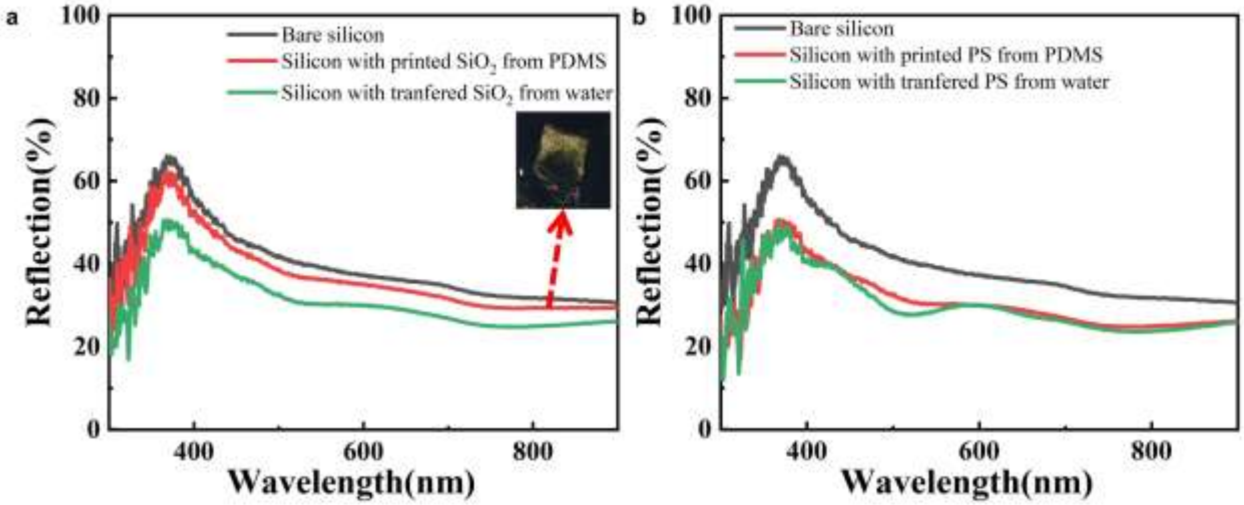

Supplementary Fig. 13. Reflectance spectra of PS and SiO_2 nanosphere films printed with 3D-NTP. a Spectra of printed and transferred SiO_2 nanospheres, and the inset shows the optical image of printed SiO_2 nanospheres, **b** Spectra of printed and transferred PS nanospheres.

The nanofilms transferred to the wafer directly using the LB transfer method can be regarded as densely packed, so the difference between the spectra of the nanospheres transferred by PDMS film without pattern and the spectra of the direct lift-on transfer also characterizes the printing efficiency. The closer the two curves are, the higher the printing efficiency is, and vice versa. It can be noticed that the two curves in Supplementary Fig. 13 differ from each, indicating the low printing efficiency of SiO_2 nanospheres (Also revealed by the inset). On the contrary, the two curves almost overlap in the case of PS nanospheres, suggesting the higher printing efficiency as discussed in the main manuscript.

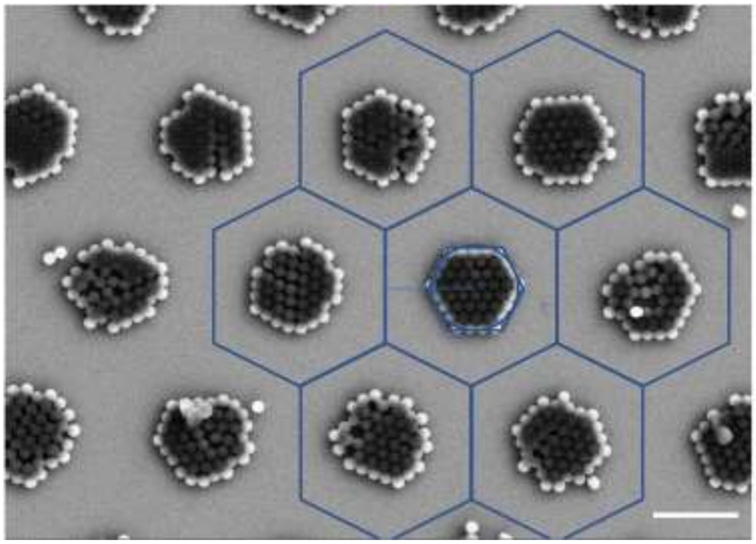

Supplementary Fig. 14. The calculation of PPI of the multiscale pattern. Scale bar, 2 µm.

We chose the smallest periodic element structure of the pattern (The blue regular hexagon with a side length of 4.04 μm in Supplementary Fig. 14) and calculated its area as 42.435 μm^2. For the constructed bud pattern with a diagonal of ~1 inch, we calculated the total area of the pattern as 3.2258 cm^2, and then divided it by the area of the smallest circular structure to find the number of pixels ~7601700. Finally, substitute it into the following formula of PPI.

$$PPI = \sqrt{\frac{\text{Resolution}}{\text{Diagonal Size (inch)}}}$$

The result of the pattern resolution is 2757 PPI.

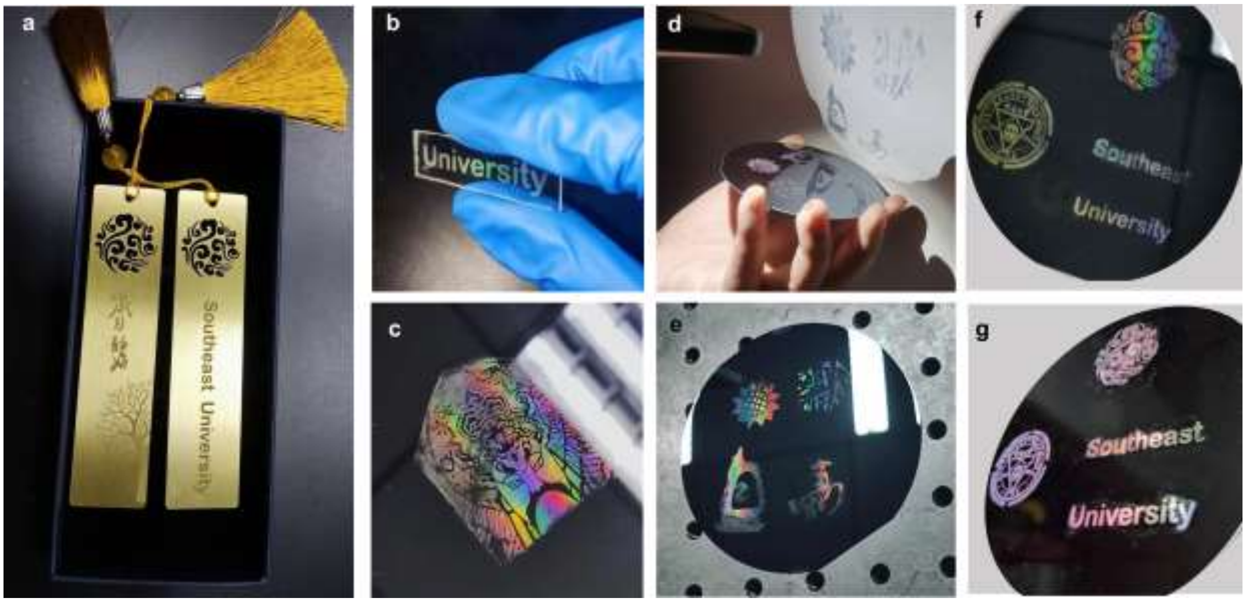

Supplementary Fig. 15. The fabrication of the multiscale patterns. a Copper intaglio. **b** The multiscale patterns on the PDMS, “University”. **c** Pattern of the Peking Opera facial makeup on a 4-inch wafer. **d** Mirror imaging of several pattern. **e** Patterns of a sunflower, bamboo forests, a sailboat, and the art Chinese character "Horse" on a 4-inch wafer. **f-g** The pattern showed brilliant color at oblique detection angles.

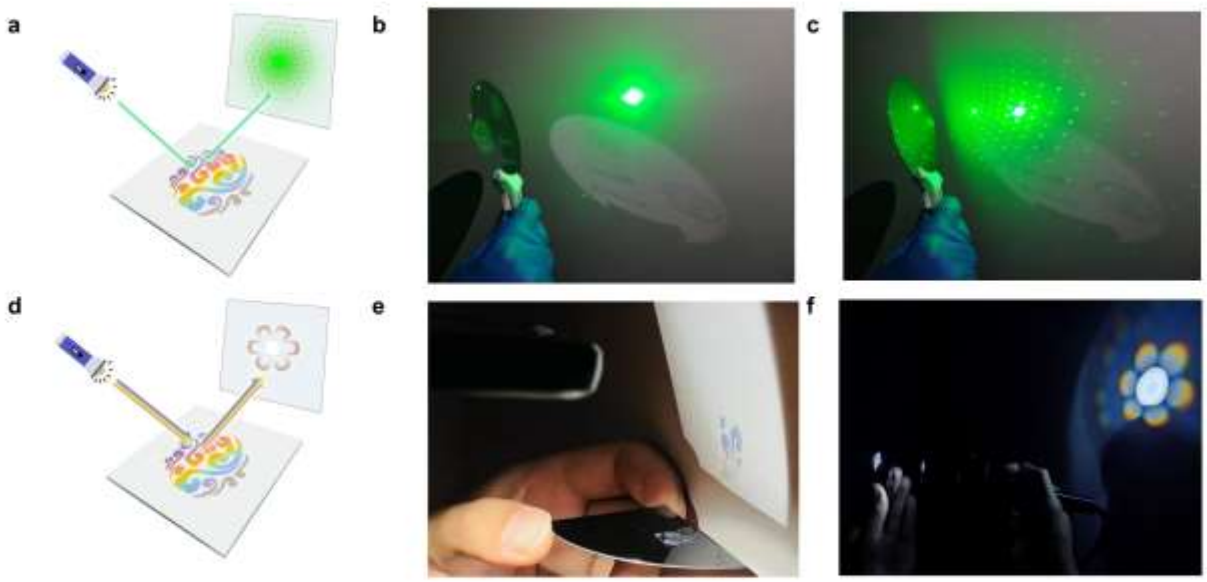

Supplementary Fig. 16. Concept of multi-modal anti-counterfeiting for the pattern “bud”. **a** Schematic diagram of laser anti-counterfeiting mode for pattern “bud”. **b** Reflective image with the green laser irradiating the blank area in the wafer. **c** Diffraction patterns with the multiscale patterns under the excitation of green laser. **d** Schematic diagram of collimated white light anti-counterfeiting mode for pattern “bud”. **e** The mirror image of multiscale patterns under omnidirectional illumination. **f** The bud became “seven-colored flowers” with the collimated white light excitation.

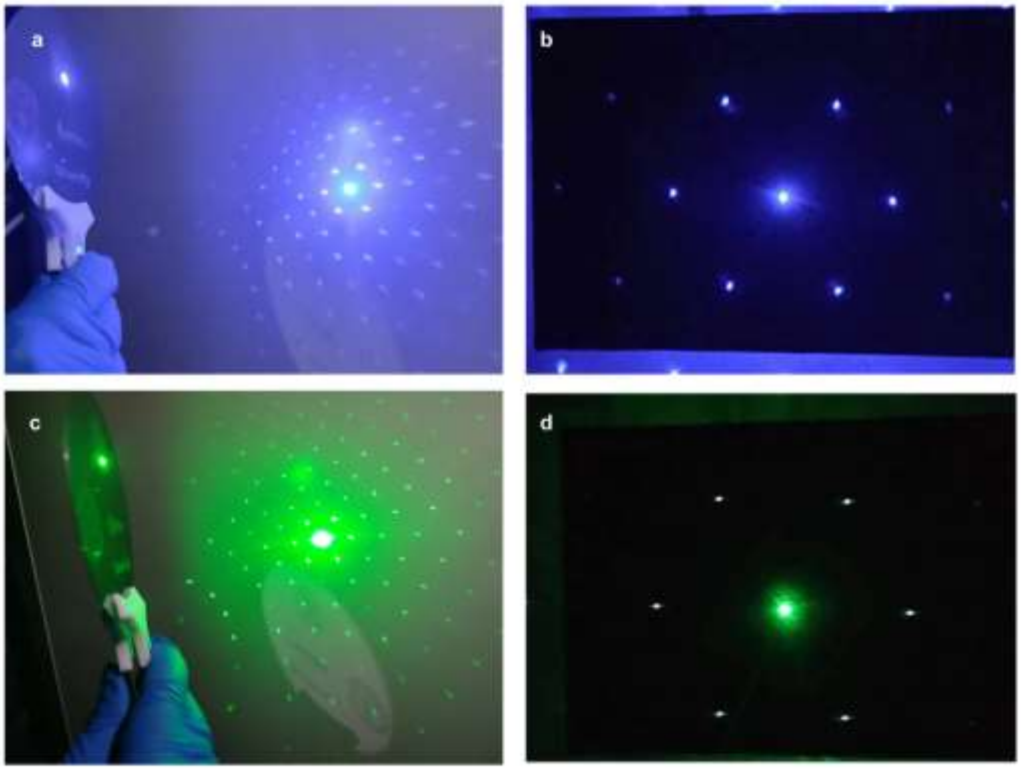

Supplementary Fig. 17. Anti-counterfeiting patterns under 450 nm and 532 nm laser. a Diffraction pattern under a 450 nm laser when the screen is ~15 cm from the substrate. **b** Diffraction pattern under a 450 nm laser when the screen is ~2 m from the substrate. **c** Diffraction pattern under a 532 nm laser when the screen is ~15 cm from the substrate. **d** Diffraction pattern under a 532 nm laser when the screen is ~2 m from the substrate. Longer screen-substrate distances clearly show the spatial variation of diffraction patterns under the excitation of different wavelengths.

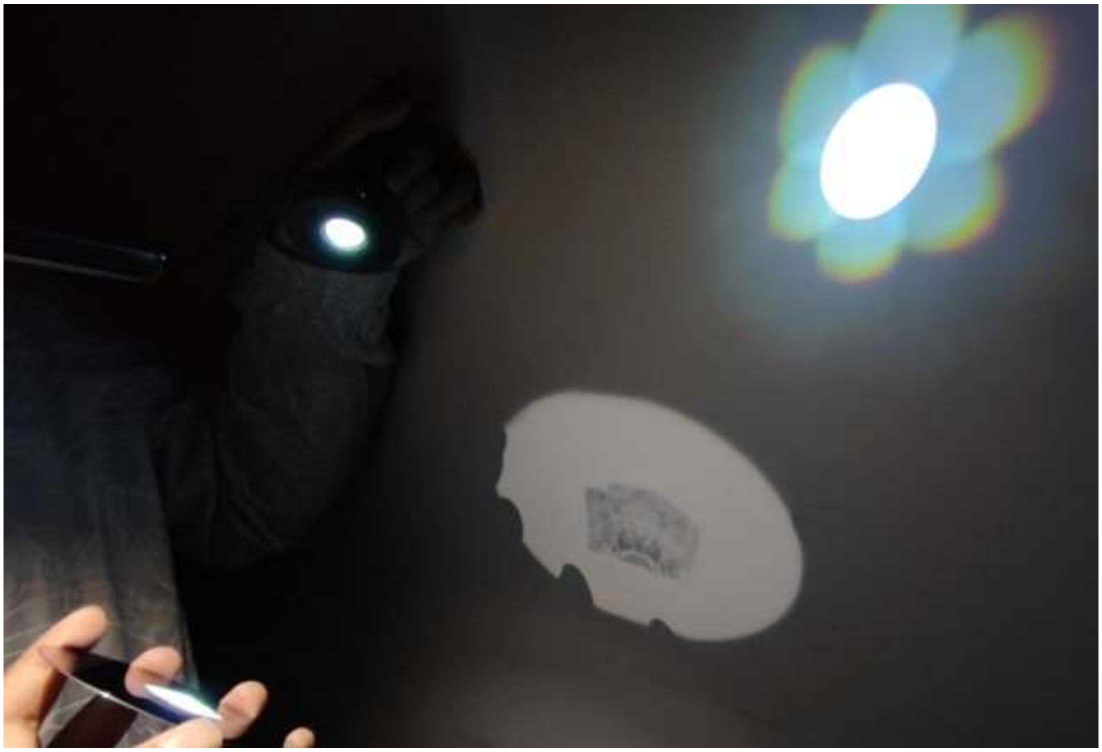

Supplementary Fig. 18. Multi-modal anti-counterfeiting under two white light sources. Mirror image under omnidirectional illumination and diffraction "flower" pattern under collimated excitation**.**

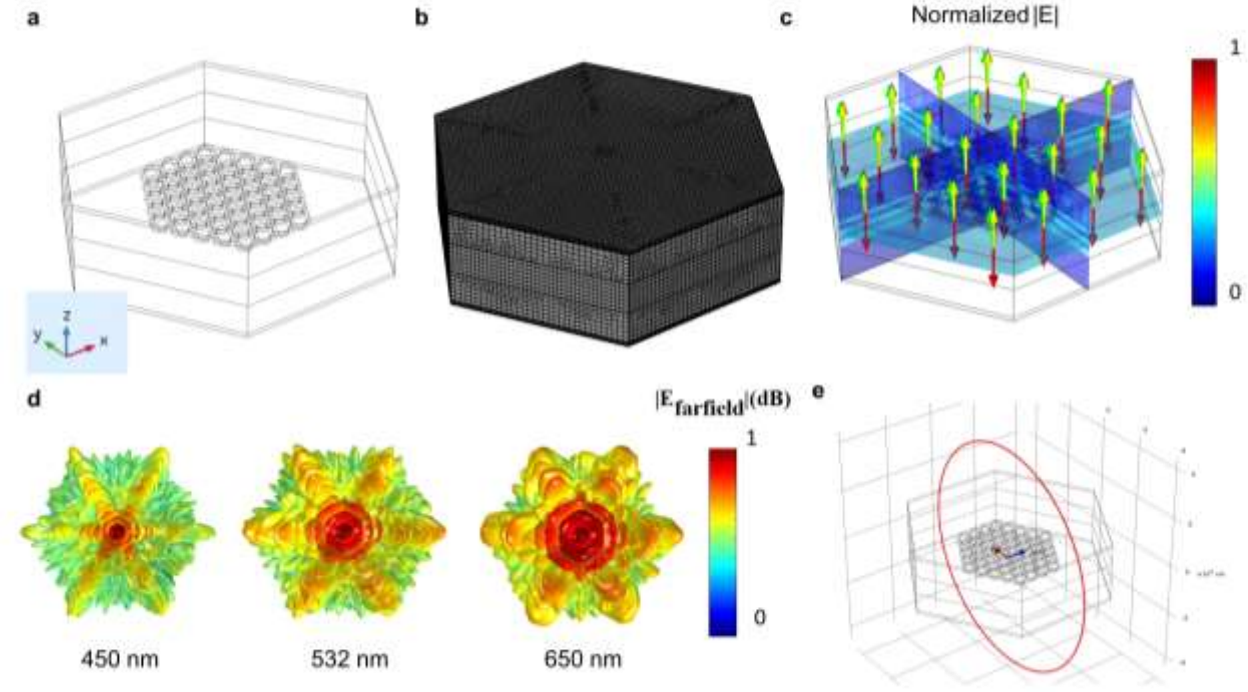

Supplementary Fig. 19. 3D finite-element simulation of the optical response of the nanosphere multiscale pattern. a Schematic diagram of the simulated nanostructure (periodic

boundary conditions). **b** Mesh division. **c** Nearfield electric field distribution. **d** Far-field scattering distribution at 450 nm, 532 nm, and 650 nm, respectively. **e** Plane (denoted by the red circle) of 2D far-field scattering simulation.

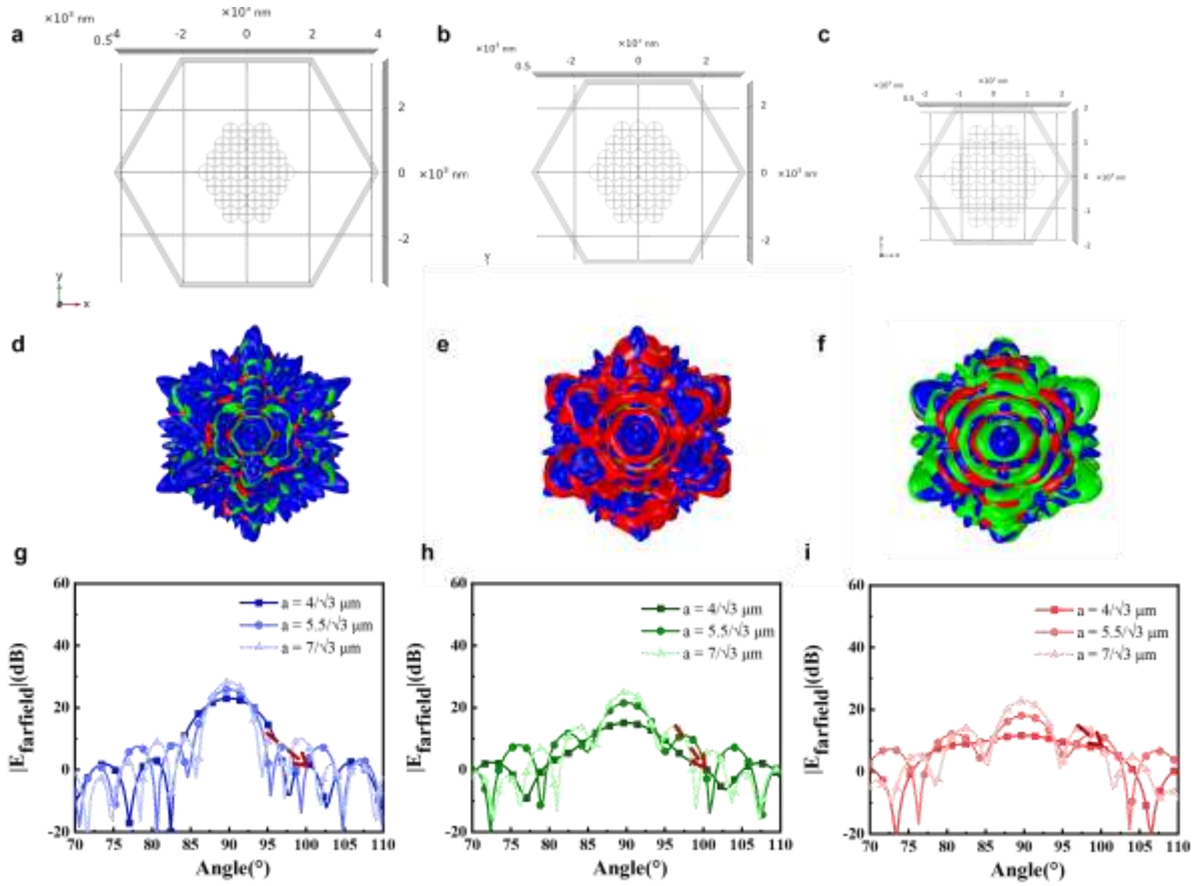

Supplementary Fig. 20. Far-field scattering calculation of the periodic patterns with different pitches of the periodic metamolecules. **a-c** The top view of a periodic unit structure. The number of the nanospheres (diameter = 500 nm) in the metamolecule was the same and set to 30. The pitch was set to 2*a* in the model, and *a* was set to $7/\sqrt{3}$ μm (**a**), $5.5/\sqrt{3}$ μm (**b**), and $4/\sqrt{3}$ μm (**c**), respectively. **d-f** 3D far-field superposition scattering pattern with excitations of 450 nm, 532 nm and 650 nm wavelengths. *a* was set to $7/\sqrt{3}$ μm (**d**), $5.5/\sqrt{3}$ μm (**e**), and $4/\sqrt{3}$ μm (**f**), respectively. **g-i** The far-field simulation (different pitch) at different detection angles under 450 nm (**g**), 532 nm (**h**), and 650 nm (**i**) excitation.

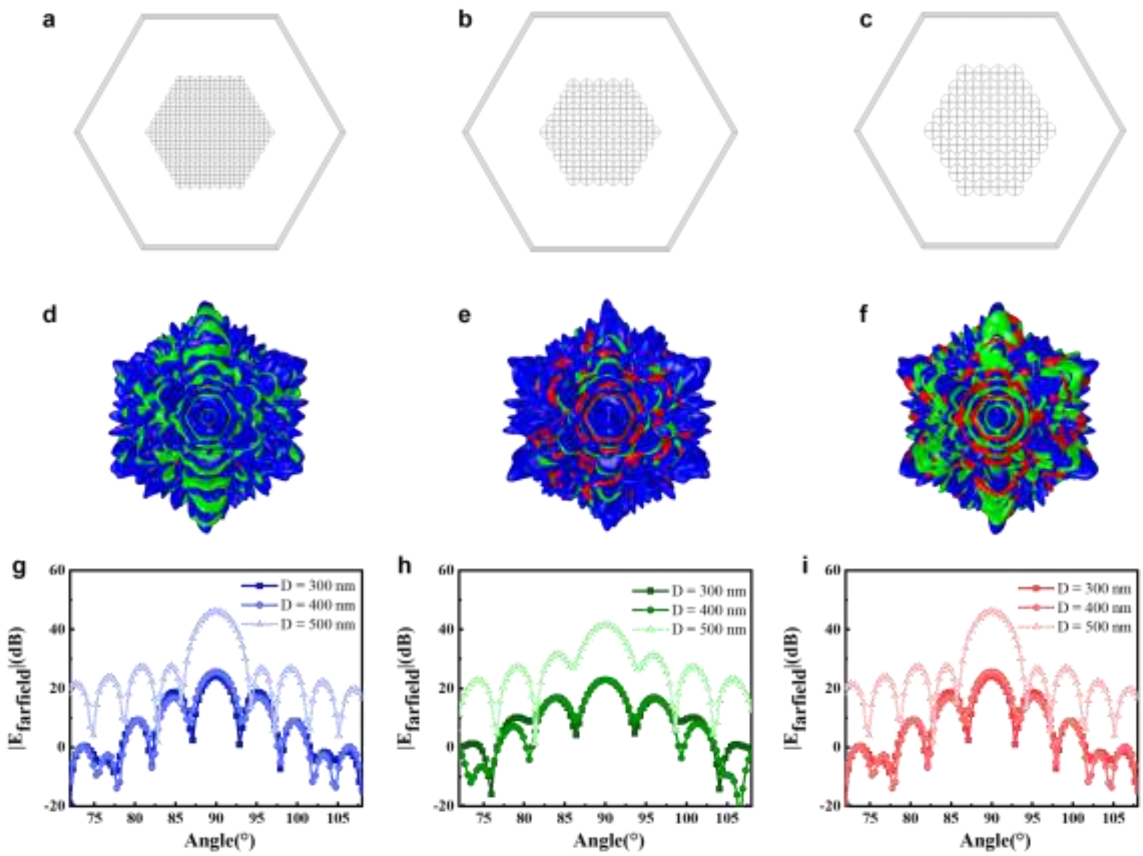

Supplementary Fig. 21. Far-field scattering calculation of the periodic patterns with different diameter (*D*) of nanospheres. **a-c** The top view of a periodic unit structure. The pitch of metamolecule was the same and set to $7/\sqrt{3}$ µm. *D* was set to 300 nm (**a**), 400 nm (**b**), and 500 nm (**c**), respectively. **d-f** 3D far-field superposition scattering pattern with excitations of 450 nm, 532 nm and 650 nm wavelengths. *D* was set to 300 nm (**d**), 400 nm (**e**), and 500 nm (**f**), respectively. **g-i** The far-field simulation (different *D*) at different detection angles under 450 nm (**g**), 532 nm (**h**), and 650 nm (**i**) excitation.

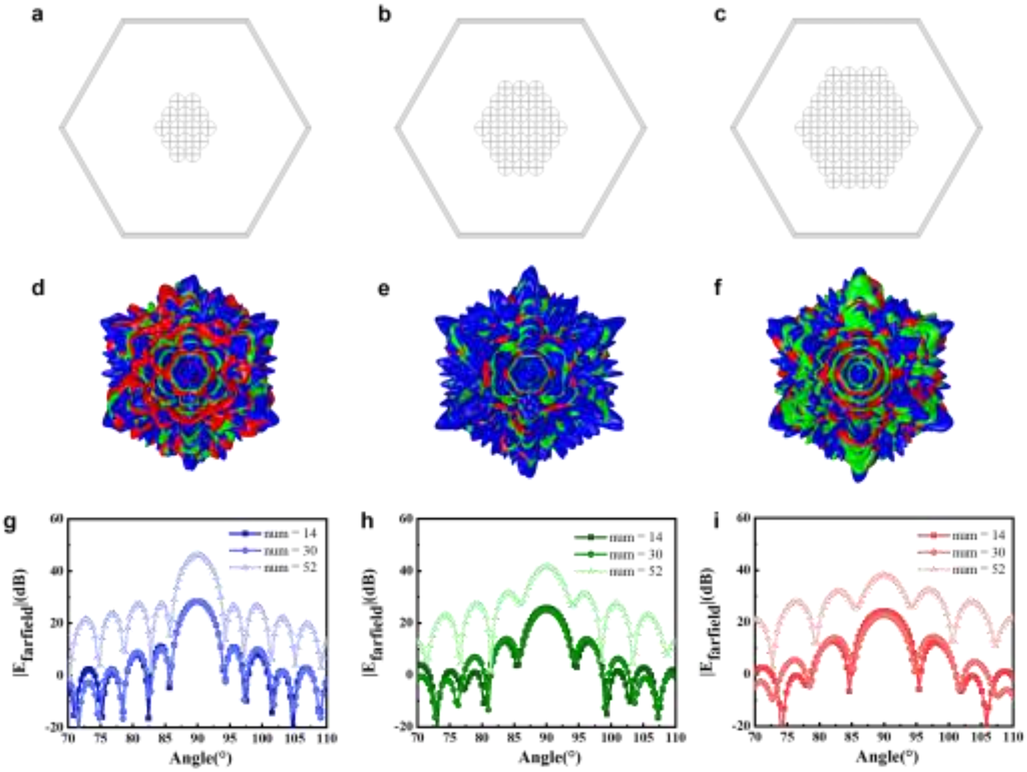

Supplementary Fig. 22. Far-field scattering calculation of the periodic patterns with different number (*num*) of nanospheres. **a-c** The top view of a periodic unit structure. The pitch of metamolecule was the same and set to $7/\sqrt{3}$ μm and nanosphere diameter was set to 500 nm. The number of the nanospheres was set to 14 (**a**), 30 (**b**), and 52 (**c**), respectively. **d-f** 3D far-field superposition scattering pattern with excitations of 450 nm, 532 nm and 650 nm wavelengths. The number of the nanospheres was set to 14 (**d**), 30 (**e**), and 52 (**f**), respectively. **g-i** The far-field simulation (different *num*) at different detection angles under 450 nm (**g**), 532 nm (**h**), and 650 nm (**i**) excitation.

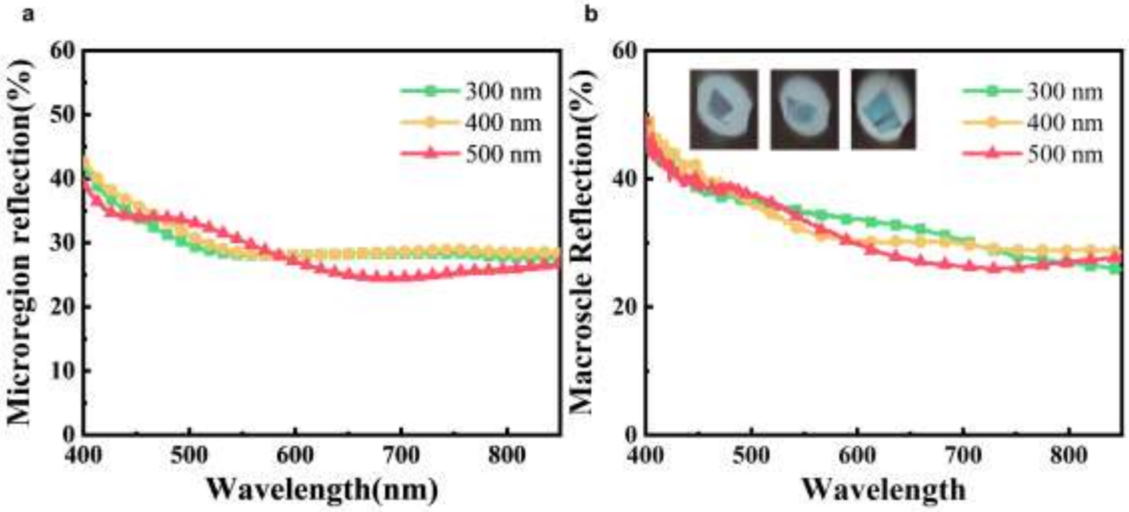

Supplementary Fig. 23. The spectra of multiscale patterns fabricated by PS nanospheres with different diameters. a The micro region spectra. **b** The macroscale spectra. The insets show

the mirror images under omnidirectional illumination. From left to right, the nanosphere diameters are 300 nm, 400 nm, and 500 nm.

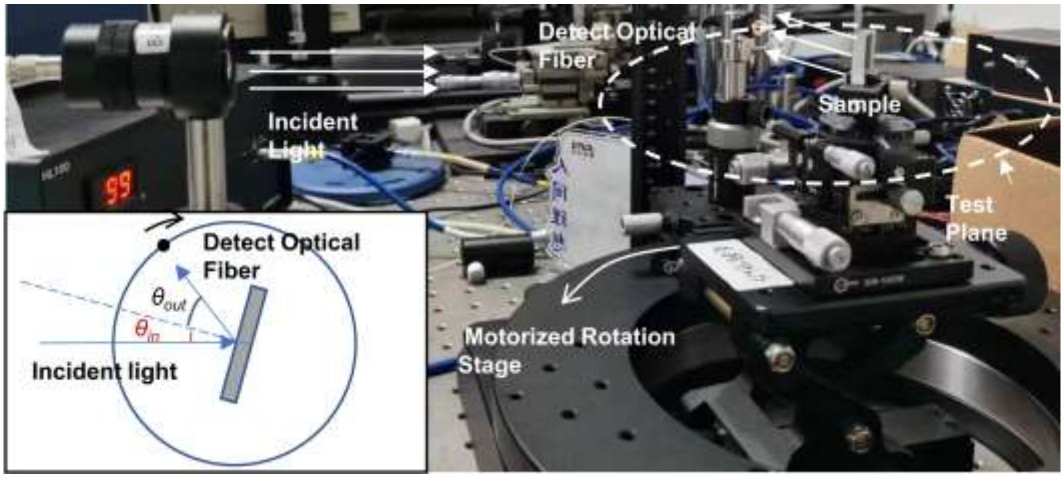

Supplementary Fig. 24. A homemade goniometric set-up.

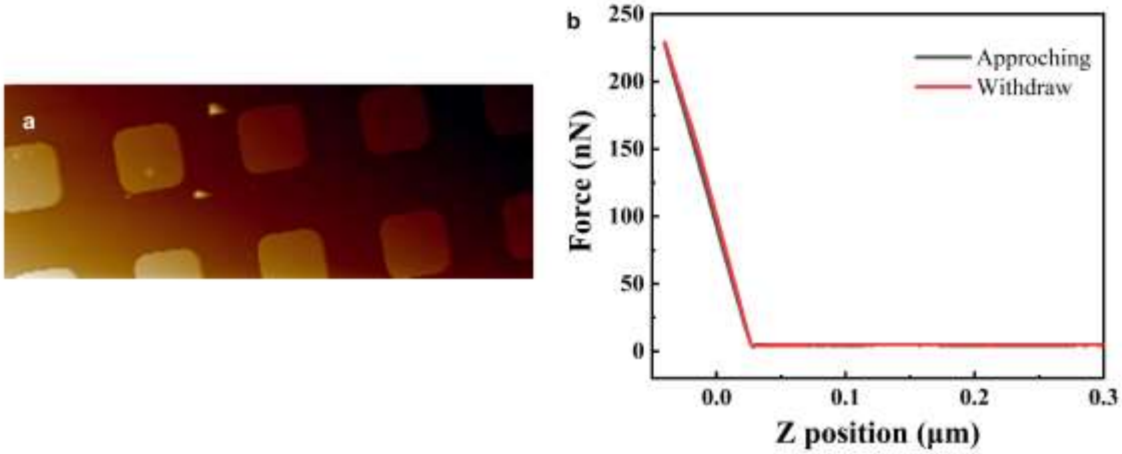

Supplementary Fig. 25. Temperature-dependent AFM calibration of Young's modulus. a Height imaging map of Al_2O_3. **b** Force curve calibration for Young's modulus calculation (k=4.291 N/m, IOS=17.277 nm/nA).

Supplementary Movie 1. Interfacial self-assembly of PS nanospheres.

Supplementary Movie 2. Laser anti-counterfeiting at randomly selected regions.

Supplementary Movie 3. Spatial locations of diffraction patterns when the screen is ~2 m from the substrate under the blue and green laser excitation.

Supplementary Movie 4. Collimated white light excitation anti-counterfeiting.
